# Arginine anchor points govern H3 tail dynamics

**DOI:** 10.3389/fmolb.2023.1150400

**Published:** 2023-05-02

**Authors:** Christine E. Jennings, Casey J. Zoss, Emma A. Morrison

**Affiliations:** ^1^ Department of Biochemistry, Medical College of Wisconsin, Milwaukee, WI, United States; ^2^ Medical Scientist Training Program, Medical College of Wisconsin, Milwaukee, WI, United States

**Keywords:** H3 tail, nucleosome core particle, protein dynamics, NMR, arginine, citrullination

## Abstract

Chromatin is dynamically reorganized spatially and temporally, and the post-translational modification of histones is a key component of this regulation. The basic subunit of chromatin is the nucleosome core particle, consisting of two copies each of the histones H2A, H2B, H3, and H4 around which ∼147 base pairs of DNA wrap. The intrinsically disordered histone termini, or tails, protrude from the core and are heavily post-translationally modified. Previous studies have shown that the histone tails exist in dynamic ensembles of DNA-bound states within the nucleosome. Histone tail interactions with DNA are involved in nucleosome conformation and chromatin organization. Charge-modulating histone post-translational modifications (PTMs) are poised to perturb the dynamic interactions between histone tails and DNA. Arginine side chains form favorable interactions with DNA and are sites of charge-modulating PTMs such as citrullination. Our current focus is on the H3 tail, the longest histone tail. Four arginine residues are relatively evenly spaced along the H3 tail sequence, suggesting multivalent interactions with DNA poised for regulation by PTMs. In this study, we use NMR nuclear spin relaxation experiments to investigate the contribution of arginine residues to H3 tail dynamics within the nucleosome core particle. By neutralizing arginine via mutation to glutamine, we begin to work towards a comprehensive understanding of the contribution of individual residues to H3 tail dynamics. We find that neutralization of arginine residues results in increased regional mobility of the H3 tails, with implications for understanding the direct effects of arginine citrullination. Altogether, these studies support a role for dynamics within the histone language and emphasize the importance of charge-modulating histone PTMs in regulating chromatin dynamics, starting at the level of the basic subunit of chromatin.

## 1 Introduction

Chromatin is dynamically reorganized during development and in response to stimuli. Dynamic regulation occurs at many length scales, and it is appealing to consider a model wherein dynamic regulation of chromosome organization and transcriptional activity originates at the level of the basic subunit, the nucleosome. The nucleosome core particle (NCP) is comprised of an octamer of histones with two copies each of the histones H3, H4, H2A, and H2B wrapped by ∼147 base pairs (bp) of DNA. The histone tails are the intrinsically disordered termini of the histones, which protrude from the nucleosome core. Histone tails have indirect and direct mechanisms of regulation, which are both modulated by post-translational modifications (PTMs). Indirect mechanisms involve the readout of histone tails and their PTMs by “reader,” “writer”, and “eraser” domains that are components of regulatory proteins and complexes (Strahl and Allis, 2000; Jenuwein and Allis, 2001). Direct mechanisms involve interactions between histone tails and DNA, which are involved in local and higher-order chromatin interactions, nucleosome stability, and accessibility to regulatory proteins (Wang and Hayes, 2007; Biswas et al., 2011; Iwasaki et al., 2013; Pepenella et al., 2013; Li and Kono, 2016; Stützer et al., 2016; Gatchalian et al., 2017; Morrison et al., 2018; Morrison et al., 2021; Peng et al., 2021).

The histone tails dynamically interact with DNA in a broad ensemble of states. Experimental characterization of nucleosomal H3 tail dynamics began with seminal work by Stützer et al. (2016) and studies to date have shown that interactions between the H3 tails and DNA are dynamic on the ps-ns and µs-ms timescales (Stützer et al., 2016; Morrison et al., 2018; Furukawa et al., 2020; Lehmann et al., 2020; Morrison et al., 2021; Zandian et al., 2021; Furukawa et al., 2022). All-atom molecular dynamics simulations have additionally shown dynamic interactions on the ns timescale (Roccatano et al., 2007; Shaytan et al., 2016; Armeev et al., 2021; Peng et al., 2021; Rabdano et al., 2021). These dynamic H3 tail-DNA interactions have thus far been shown to be altered by nucleosome assembly state, the additional presence of linker DNA, binding of H1 to linker DNA, incorporation into nucleosome arrays, partial DNA replacement by FACT, charge-modulating mutations in the H2A core, select serine phosphorylation, and select lysine acetylation on the H3 and H4 tails (Gao et al., 2013; Stützer et al., 2016; Furukawa et al., 2020; Lehmann et al., 2020; Pelaz et al., 2020; Tsunaka et al., 2020; Morrison et al., 2021; Zandian et al., 2021; Furukawa et al., 2022). Histone tail accessibility to regulatory proteins/complexes correlates with dynamics, where an increase in ps-ns dynamics is associated with an increase in binding, enzymatic, or chemical reactivity on the tail (Stützer et al., 2016; Gatchalian et al., 2017; Tsunaka et al., 2020; Morrison et al., 2021). This correlation supports a functional role for histone tail dynamics within direct mechanisms of histone-tail-based regulation.

Is the modulation of histone tail dynamics by PTMs a regulatory mechanism within the histone language? Charge-modulating histone PTMs, which include acylation of lysine, citrullination of arginine, and phosphorylation of serine and threonine, are prime candidates for modulating histone tail interactions with DNA and thus histone tail dynamics. Our overall goal is to decipher the role of dynamics in the histone language. This involves characterizing the dynamics of the multivalent network between histone tail residues and DNA by cataloging each tail residue and the effect of mutating or modifying the residue. It is interesting to consider whether there is long-range communication between residues via dynamics, which would be a form of histone PTM crosstalk. Histone tails are rich in both arginine and lysine, but the arginine side chain is particularly favorable for interactions with DNA, especially within a narrowed minor groove (Rohs et al., 2009). The H3 tail has arginine at positions 2, 8, 17, and 26, relatively evenly distributed along the length of the 36-aa tail. As a first step toward a comprehensive understanding of histone tail dynamics at residue resolution and modulation by modifications, we conducted a systematic study of the effect of H3 tail arginine neutralization on H3 tail dynamics within the NCP.

In this study, we use NMR spin relaxation to measure the ps-ns timescale motions of the H3 tail within the NCP. Neutralization of arginine via mutation to glutamine leads to an increase in dynamics on this timescale with neutralization of all four H3 tail arginines resulting in a global increase across the tail. Neutralization of each individual arginine leads to a regional increase with the extent of the effect dependent on the arginine position. Notably, the increase in dynamics is purveyed across ∼60%–70% of the tail for R8Q, R17Q, and R26Q at 150 mM KCl. In general, the largest effect is observed for neutralization of R26, adjacent to the putative H3 tail ‘hinge’. This study provides insight into mechanisms of histone PTM crosstalk and tail accessibility and additionally has implications for the regulatory mechanisms of citrullination.

## 2 Materials and methods

### 2.1 Preparation of histones and DNA

Histones were expressed and purified as previously described (Dyer et al., 2004; Morrison et al., 2018; Paintsil and Morrison, 2022). The human histone sequences used were: H2A (UniProt P0C0S8), H2B (UniProt P62807), H3 (UniProt Q71DI3 with C110A), and H4 (UniProt P62805). Mutants of histone H3 (R2Q, R8Q, R17Q, R26Q, and R2/8/17/26Q) were generated via site-directed mutagenesis. Wild-type and mutant H3 were isotopically-labeled via expression in ^15^N-M9 minimal media. To confirm histone masses, electrospray ionization mass spectrometry was performed. 147 bp Widom 601 DNA was amplified and purified as previously described (Dyer et al., 2004; Paintsil and Morrison, 2022). The DNA sequence is ATC​GAG​AAT​CCC​GGT​GCC​GAG​GCC​GCT​CAA​TTG​GTC​GTA​GAC​AGC​TCT​AGC​ACC​GCT​TAA​ACG​CAC​GTA​CGC​GCT​GTC​CCC​CGC​GTT​TTA​ACC​GCC​AAG​GGG​ATT​ACT​CCC​TAG​TCT​CCA​GGC​ACG​TGT​CAG​ATA​TAT​ACA​TCC​GAT.

### 2.2 Reconstitution of nucleosome core particles

NCPs were reconstituted as previously described (Dyer et al., 2004; Hammonds and Morrison, 2022). Briefly, ^15^N-H3/H4 tetramer and H2A/H2B dimer were refolded separately by dialysis from denaturing conditions into 2 M KCl. Refolded histones and DNA were mixed at a 1:1:2.2 molar ratio of DNA:^15^N-H3/H4 tetramer:H2A/H2B dimer and slowly desalted via an exponential gradient to form NCPs. Reconstituted NCPs were purified via 10%–40% sucrose gradient. Sucrose gradient profiles along with 5% native and 18% SDS polyacrylamide gel electrophoresis were used to assess the quality of the samples.

### 2.3 NMR sample preparation

Purified NCPs were exchanged into NMR buffer using consecutive rounds of concentration and dilution in 10k MWCO centrifugal filtration devices. NCP concentration was determined spectrophotometrically via absorbance at 260 nm. Samples were first diluted into 2 M salt, and concentrations were determined using the calculated extinction coefficient for the DNA (*ε*_260_ = 2,312,300.9 M^-1^cm^−1^). Final samples for data collection were 100 µM ^15^N-H3 NCP in 20 mM MOPS pH 7 (with 8 mM NaOH to pH), 1 mM EDTA, 1 mM DTT, and 5% D_2_O. Samples additionally contained 150 mM KCl where noted.

### 2.4 NMR nuclear spin relaxation data collection

Data were collected on a Bruker Avance Neo 800 MHz spectrometer with cryogenic probe at 304 K and running Topspin 4.0.7. For chemical shift perturbation analysis, ^1^H-^15^N HSQC spectra were collected with 24 scans, 2,048 (^1^H) × 400 (^15^N) total points, acquisition times of 98 ms (^1^H) and 82 ms (^15^N), and spectra widths of 13 ppm (^1^H) and 30 ppm (^15^N). All nuclear spin relaxation experiments were collected with 2,048 (^1^H) × 300 (^15^N) total points, acquisition times of 98 ms (^1^H) and 84 ms (^15^N), and spectra widths of 13 ppm (^1^H) and 22 ppm (^15^N). {^1^H}-^15^N steady-state heteronuclear nuclear Overhauser effect (hnNOE) data were collected using the Bruker pulse sequence hsqcnoef3gpsi with the reference and saturated spectra interleaved. Parameters included 96 scans with an interscan delay of 5 s. Longitudinal (R1) ^15^N relaxation experiments were collected with total relaxation loop lengths of 0.033 s, 0.195, 0.390 s (×2), 0.813 s (×2), 1.301 and 1.951 s in randomized order. Experiments were collected with 32 scans and an interscan delay of 2 s. Transverse (R2) ^15^N relaxation experiments were collected with an effective CPMG field of 500 Hz and total relaxation CPMG loop lengths of 0.0088, 0.031 s (×2), 0.053, 0.079, 0.110 s (×2), 0.150 s, with the exception of the wild-type H3-NCP in 150 mM KCl, which was collected with delay times of 0.0044, 0.018 s (×2), 0.040, 0.062 s, 0.084 s (×2), and 0.110 s. Experiments included temperature compensation blocks and were collected in randomized order, with 32 scans, and with an interscan delay of 2.5 s. HSQC spectra were collected for each sample between relaxation series to confirm sample integrity.

### 2.5 NMR data analysis

NMR data were processed using NMRPipe (Delaglio et al., 1995) and analyzed using CcpNmr Analysis 2.5.2 (Vranken et al., 2005). NMRbox was used for processing and analysis (Maciejewski et al., 2017). Data were plotted in Igor Pro (Wavemetrics). Assignments were taken from BMRB entry 50806 for NCPs with wild type H3. Amide assignments were transferred to H3 mutants via inspection and comparison of ^1^H-^15^N HSQC spectra of all mutant H3-NCP. Questionable assignment transfers at 0 mM KCl are as follows: K9 and K18 for ^15^N-R17Q-H3-NCP and L20 and K23 for ^15^N-R17Q- and R2/8/17/26Q-H3-NCP. Questionable assignment transfers at 150 mM KCl are as follows: K9 and K18 for ^15^N-R2/8/17/26Q-H3-NCP and L20 and K23 for all constructs. The assignment for R2 was used from Furukawa et al. (2020). The following peaks were analyzed as doublets at 0 mM KCl (grouped for each sample): WT (R2, K36), R2Q (K36), R8Q (R2, T3, Q5, K36), R17Q (R2, Q5, T6, R8, K9, L20, K36), and R26Q (R2, Q5, T6, R8). Chemical shift perturbations (CSPs, ∆δ ) between wild-type and mutant ^15^N-H3-NCP were calculated from the differences in amide proton ( ${∆\delta }_{H}$ ) and amide nitrogen ( ${∆\delta }_{N}$ ) chemical shifts using: $∆\delta =\sqrt{{\left({∆\delta }_{H}\right)}^{2}+{\left(0.154{∆\delta }_{N}\right)}^{2}}$ (Tugarinov and Kay, 2003). For calculation of CSPs with peak doublets, if the peak for a given residue in WT-H3-NCP was a singlet, then the CSP of both mutant peaks was calculated with respect to the single WT peak. Both values are plotted in the corresponding figures.

Spectra from relaxation experiments were processed using a cosine-squared window function for both ^1^H and ^15^N dimensions. Each dimension was doubled in size by zero-filling twice, rounding to the nearest power of two. HnNOE values for each residue were calculated as the ratio of peak intensity (peak height) from the saturated and reference spectra, and errors were calculated via standard error propagation of the spectral noise as determined by CcpNmr Analysis 2.5.2. Relaxation rates (R1 and R2) were determined by fitting peak heights from the relaxation series to a single-exponential decay without offset, and errors were determined from the covariance matrix within CcpNmr Analysis 2.5.2. The R2/R1 ratio was calculated from the fit values for R1 and R2, and error was determined using standard error propagation. As is common with intrinsically disordered regions, amide spectra suffer from low chemical shift dispersion. These residues were analyzed in the same manner described and are included in the overall analysis with the caveat that the relaxation rates determined from the analysis are likely influenced by convolution with neighboring peak(s). In order to test the robustness of the analysis of overlapping peaks, a separate analysis was conducted for ^15^N-WT-H3-NCP at 0 mM KCl using PINT v2.1.0 (Ahlner et al., 2013; Niklasson et al., 2017), which directly fits overlapping line shapes. Peak integration was performed using the galore line shape with peak radii of 0.3 and 0.06 ppm for ^15^N and ^1^H, respectively. Errors in R1 and R2 were determined via jackknife error analysis. Similar results from CcpNmr Analysis and PINT support a robust analysis (Supplementary Figure S1).

Residues with peak overlap are denoted in Supplementary Figures S2, S3. For data collected at 0 mM KCl, these residues are as follows (grouped for each sample): WT (K9, R17, K18, Q19, L20, K23), R2Q (K9, R17, K18, Q19, L20, K23, K36), R8Q (T3, Q5, A7, R17, Q19, L20, K23, K36), R17Q (Q5, T6, A7, R8, K9, K18, L20, K23, K36), R26Q (Q5, T6, A7, R8, K9, K18, L20, K23, K36), and R2/8/17/26Q (K9, K18, K36). For data collected at 150 mM KCl, these residues are as follows (grouped for each sample): WT (K9, R17, K18, Q19, L20, K23), R2Q (K9, R17, K18, Q19, L20, K23), R8Q (K9, G12, G13, R17, K18, Q19, L20, K23, K27, G33, G34), R17Q (K9, G13, K18, L20, K23, K27, G34), R26Q (K9, R17, K18, Q19, L20, K23, K36), and R2/8/17/26Q (K9, G12, G13, K18, G33, G34, K36). The following residues were omitted from analysis. L20 was omitted from relaxation analysis in WT-, R2Q-, R8Q-, and R26Q-H3-NCP at 0 mM KCl and WT at 150 mM KCl. R2 was omitted from relaxation analysis in all samples at 0 mM KCl and was not visible at 150 mM KCl. K36 was omitted from relaxation analysis in all samples at 150 mM KCl.

The difference in hnNOE and R2/R1 ratio, ΔhnNOE and Δ(R2/R1), was calculated for each mutant with respect to WT. For calculation of ΔhnNOE and Δ(R2/R1) with peak doublets, if the peak for a given residue in WT-H3-NCP was a singlet, then the ΔhnNOE or Δ(R2/R1) of both mutant peaks was calculated with respect to the single WT peak. Both values are plotted in the corresponding figures.

## 3 Results

### 3.1 Baseline dynamics of the H3 tail within NCP

**FIGURE 1:**
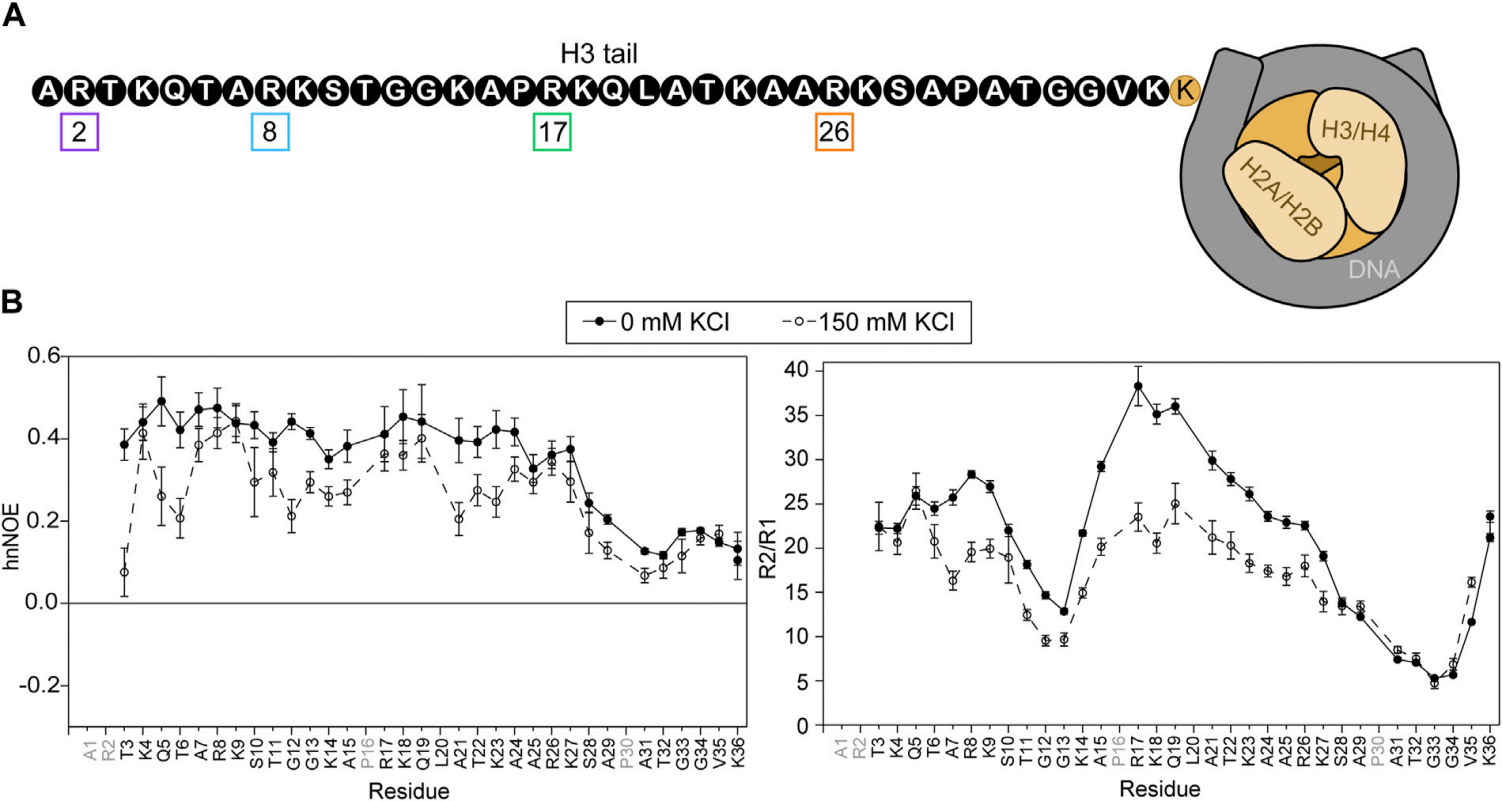
Basal state of the H3 tail within the NCP. **(A)** A cartoon depiction of the NCP shows a complex of two H3/H4 dimers and two H2A/H2B dimers (shades of beige) wrapped by DNA (grey). The H3 tail sequence is explicitly depicted (not to scale) in black and white. Arginine residues are numbered and boxed in colors corresponding to plots in subsequent figures. **(B)** Amide nuclear spin relaxation data is shown for ^15^N-WT-H3-NCP in the absence (closed circles, solid line) and presence (open circles, dashed line) of 150 mM KCl. HnNOE (left) and R2/R1 (right) are plotted as a function of H3 tail residue. Two values are plotted for doublet peaks (see Section 2). Error bars represent standard error propagation of the spectral noise for hnNOE values and were determined via the covariance matrix in fitting R1 and R2 decay curves, which was subsequently propagated for R2/R1. Data were collected at 800 MHz and 304 K.

It has previously been established that, although the NCP is ∼200 kDa in size, the histone tails are visible via backbone amide-detected solution NMR spectroscopy due to the mobility of these intrinsically disordered regions (IDRs) (Zhou et al., 2012). The H3 tail dynamically interacts with DNA within the nucleosome and NCP in a broad ensemble of conformations (Stützer et al., 2016; Morrison et al., 2018), and adding monovalent salt globally increases the dynamics of the tail (Furukawa et al., 2020). Here, we start by confirming these results under our experimental conditions and at 0 or 150 mM added KCl to establish baseline values for comparison of the H3 tail arginine mutants (Figures 1A, B; Supplementary Tables S1, S2). NCPs were reconstituted with ^15^N-labeled WT-H3 along with unlabeled H2A, H2B, and H4 and 147 bp Widom 601 DNA. Consistent with previous results, H3 is only visible in amide spectra through K36 (Zhou et al., 2012; Stützer et al., 2016; Morrison et al., 2018; Furukawa et al., 2020; Zandian et al., 2021). A1, P16, and P30 are by nature not visible, and R2 and L20 are only weakly visible in ^1^H-^15^N HSQC spectra, precluding them from analysis.

^15^N-WT-H3-NCP was subjected to NMR spin relaxation experiments, which provide insight into nucleosomal H3 tail mobility on the ps-ns timescale (Figure 1B). HnNOE values for the H3 tail within WT-H3-NCP vary between 0.10 and 0.49, with an average value of 0.34 ± 0.12. Average R1 and R2 relaxation rates along the tail are 1.07 ± 0.09 s^−1^ and 22 ± 8 s^−1^, respectively, and the average R2/R1 ratio is 21 ± 9. The R2/R1 ratio is related to the effective rotational correlation time for each residue and includes contributions from overall tumbling in addition to internal molecular motions. The hnNOE profile for the nucleosomal H3 tail (WT) shows a decrease in hnNOE values for residues S28-K36 (average = 0.16 ± 0.05) as compared to residues T3-K27 (average = 0.41 ± 0.04), supporting a putative flexible ‘hinge’ region. As noted initially by Stützer et al. (2016) there are two TGG repeats (residues 11–13 and 32–34) in the H3 tail. Both regions display dips in the hnNOE and/or R2/R1 profiles, but the second repeat is accompanied by a larger swath of uncharged residues and is correspondingly wider and deeper in these profiles. The trends described above support a shorter effective rotational correlation time and thus greater mobility on the ps-ns timescale in the two TGG repeats as compared to the remainder of the H3 tail, which extends into a more extensive ‘hinge’ region in residues ∼S28-K36. This flexible ‘hinge’ has previously been noted (Furukawa et al., 2020; Morrison et al., 2021; Tsunaka et al., 2022). As a point of clarification regarding the difference between hnNOE and R2/R1 for V35 and K36, overall particle tumbling likely starts dominating near the core for R2/R1, leading to an observed decrease in ps-ns mobility at the C-terminal end of tail ‘hinge’. The differential influence of overall tumbling may also lead to a dip in the R2/R1 profile for the first TGG repeat, not seen in the hnNOE. The addition of 150 mM KCl leads to decreases in hnNOE and R2/R1 across the H3 tail. There is overall more heterogeneity in hnNOE values for ^15^N-WT-H3-NCP across residues T3-K27 (average hnNOE = 0.30 ± 0.08) as compared to at 0 mM KCl when considering the standard deviation. The putative ‘hinge’ region is maintained for S28-K36 but is less distinct than at 0 mM KCl (average hnNOE = 0.13 ± 0.04). Altogether, these data support a model, in agreement with the literature, wherein the H3 tails are neither conformationally rigid nor unrestrained but rather are conformationally restrained by dynamic interactions with DNA.

### 3.2 Chemical shift perturbations resulting from arginine-neutralizing mutations are largely localized

In order to catalog the effects of neutralizing the four H3 tail arginine residues, both individually and all together, on H3 tail dynamics within the NCP, we used NMR spectroscopy. We reconstituted NCPs with ^15^N-labeled mutants of H3, either one of four single-arginine mutants of H3 (R2Q, R8Q, R17Q, or R26Q) or a quadruple-arginine mutant of H3 (R2/8/17/26Q). Amide assignments were transferred from WT to each of the four single-arginine mutants of H3 via inspection, and the single-arginine mutants were together used to transfer amide assignments to R2/8/17/26Q-H3.

A subset of residues is split into two peaks, which is dependent on the H3 construct used, and additional peaks appear broadened (Supplementary Figure S2). At 304 K and 0 mM KCl, two peaks are seen for: R2 and K36 for WT-H3; K36 for R2Q-H3; R2, T3, Q5, and K36 for R8Q-H3; R2, Q5, T6, R8, K9, L20, and K36 for R17Q-H3; and R2, Q5, T6, and R8 for R26Q-H3. The broadened and split peaks have been observed previously (Morrison et al., 2021) and may be due to asymmetry in the NCP resulting from asymmetry in the DNA sequence.

**FIGURE 2:**
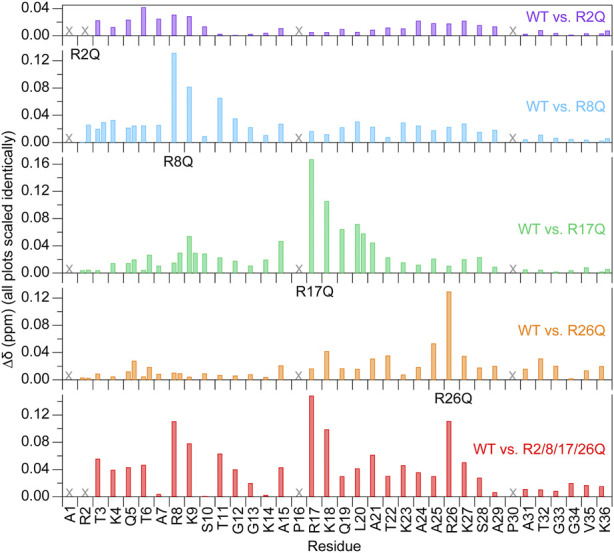
Chemical shift perturbations are localized near the mutated arginine at 0 mM KCl. Chemical shift perturbations ( ∆δ ) are plotted as a function of H3 tail residue for ^1^H-^15^N HSQC spectra of each mutant as compared to WT-H3-NCP at 0 mM KCl. Two values are plotted for doublet peaks (see Section 2). “X” symbols mark residues that are not visible. Data were collected at 800 MHz and 304 K.

Due to the sensitivity of chemical shifts to conformation, chemical shift perturbations (CSPs) determined from ^1^H-^15^N HSQC spectra were used for an initial evaluation of the effects of the arginine-neutralizing mutations on the conformational ensemble of the H3 tail within the NCP (Figure 2). CSPs of all non-mutated residues are less than or equal to 0.1 ppm, and, with few exceptions, CSPs of residues more than five positions away from mutated arginines are under 0.04 ppm. These low-magnitude perturbations are generally common for IDRs, including for the H3 tail, which shows few perturbations that exceed 0.1 ppm even between apo and DNA-bound peptide forms (Morrison et al., 2018). While the largest CSPs are generally restricted to the local vicinity of the mutated arginine, there are some remote variations. For example, see residues near R26 when either R2 or R8 is mutated. These perturbations could suggest longer-range effects on the H3 tail conformational ensemble. For the R2Q and R8Q mutations, the majority of peak shifts follow the rough trajectory between a peptide version of the H3 tail and NCP/nucleosome (Stützer et al., 2016; Morrison et al., 2018), moving towards this unbound version of the tail as might be expected for perturbing a DNA-bound conformational ensemble with charge-neutralizing mutations. Interestingly, this was not the case for R17Q and R26Q mutations.

### 3.3 Neutralizing the four H3 tail arginine residues leads to a global increase in H3 tail mobility

**FIGURE 3:**
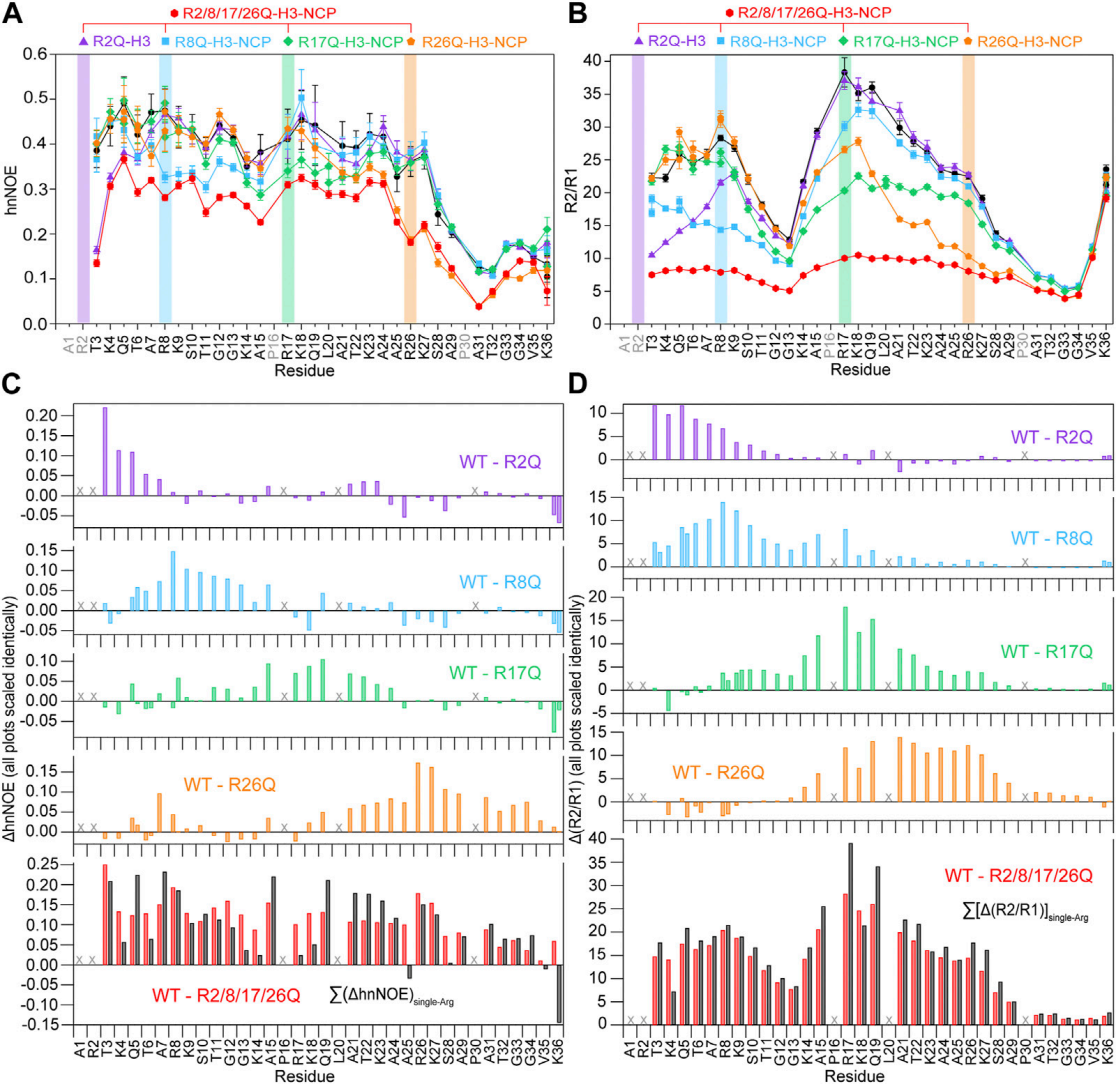
Neutralizing H3 tail arginines leads to a regional increase in H3 tail dynamics at 0 mM KCl. Plots are shown for **(A)** hnNOE values and **(B)** R2/R1 as a function of residue for WT- (black circles), R2Q- (purple triangles), R8Q- (blue squares), R17Q- (green diamonds), R26Q- (orange pentagons), and R2/8/17/26Q-H3-NCP (red hexagons). Error bars represent standard error propagation of the spectral noise for hnNOE values and were determined via the covariance matrix in fitting R1 and R2 decay curves, which was subsequently propagated for R2/R1. Note that some error bars are smaller than the symbols. Difference plots for **(C)** hnNOE and **(D)** R2/R1 show the difference between the per-residue hnNOE or R2/R1 for the H3 tail within ^15^N-WT-H3-NCP and within each single-arginine mutant (top four plots). The bottom plots show the difference between WT- and R2/8/17/26Q-H3-NCP (red) and the sum of the four difference plots for each single-arginine mutant (i.e., ΔhnNOE_WT-R2Q_ + ΔhnNOE_WT-R8Q_ + ΔhnNOE_WT-R17Q_ + ΔhnNOE_WT-R26Q_ on the lower left and Δ(R2/R1)_WT-R2Q_ + *Δ*(R2/R1)_WT-R8Q_ + *Δ*(R2/R1)_WT-R17Q_ + *Δ*(R2/R1)_WT-R26Q_ on the lower right) (black). “X” symbols mark residues omitted from analysis. Two values are plotted for doublet peaks (see Section 2). Data were collected at 0 mM KCl, 800 MHz, and 304 K.

In order to determine the greatest potential impact of arginine neutralization on H3 tail dynamics, we started by comparing ^15^N-WT-H3-NCP and ^15^N-R2/8/17/26Q-H3-NCP. NMR spin relaxation experiments reveal differences between H3 tail dynamics on the ps-ns timescale with neutralization of all four tail arginine residues. The hnNOE value for the H3 tail within R2/8/17/26Q-H3-NCP varies between 0.04 and 0.37 (average = 0.24 ± 0.09) at 0 mM KCl, an average decrease from WT of 31% (Figure 3A; Supplementary Table S1). In line with the hnNOE data, R2/8/17/26Q-H3-NCP has an average increase in ^15^N R1 of 17% (average R1 = 1.25 ± 0.05 s^−1^), decrease in ^15^N R2 of 54% (average R2 = 10 ± 3 s^−1^), and decrease in R2/R1 ratio of 62% (average R2/R1 = 8 ± 3) across the H3 tail as compared to WT. Together, these trends support an overall increase in H3 tail dynamics on the ps-ns timescale as a result of neutralizing mutations to all four arginine residues. Despite the overall increase in mobility as compared to WT, hnNOE values for H3 tail residues S28-K36 within R2/8/17/26Q-H3-NCP remain depressed (average = 0.11 ± 0.04) as compared to residues T3-K27 (average = 0.28 ± 0.05), a trend that was observed for WT. Similarly, the R2/R1 profile shows smaller values for S28-K36 and additionally around the first TGG repeat at residues 11–13 (Figure 3B; Supplementary Table S1).

Altogether, the hnNOE and R2/R1 profiles indicate a global increase in H3 tail dynamics on the ps-ns timescale as a result of glutamine mutations to all four arginine residues. We interpret these data to mean that neutralization of H3 tail arginine residues increases tail dynamics by eliminating electrostatic interactions with DNA at those points. The H3 tail retains a more flexible ‘hinge’ region in residues S28-K36 even as the overall ps-ns motions increase. Notably, H3 tail lysines remain, which would still provide interaction points with DNA outside of the TGG repeats and ‘hinge’.

### 3.4 Neutralizing each of the four H3 tail arginine residues individually leads to regional increases in H3 tail mobility

We next asked whether neutralizing an individual H3 tail arginine residue has an effect on tail dynamics, whether this effect is localized or more extensive (i.e., regional or global), and whether there is a positional effect within the tail (i.e., whether each arginine position has the same effect). NMR nuclear spin relaxation experiments collected on NCPs reconstituted with ^15^N-labeled R2Q-, R8Q-, R17Q-, or R26Q-H3 were compared with data from ^15^N-WT-H3-NCP and ^15^N-R2/8/17/26Q-H3-NCP. As a point of clarification, residue A1 is not observable and R2 is only weakly observed in ^1^H-^15^N HSQC spectra and not of sufficient intensity for analysis of nuclear spin relaxation experiments, but residues T3 and greater still provide insight into the effect of the R2Q mutation.

While less extensive than with neutralization of all four tail arginine residues together, nuclear spin relaxation experiments reveal differences in H3 tail dynamics on the ps-ns timescale with neutralization of each individual H3 tail arginine (Figure 3; Supplementary Figures S4, S5; Supplementary Table S1). Each mutation at a minimum increases the local ps-ns dynamics as seen by a decrease in hnNOE value and R2/R1 ratio in the vicinity of the mutation. We used several approaches to compare and rank the effect of each mutation on ps-ns dynamics. Difference plots of hnNOE and R2/R1 ratio, ΔhnNOE and Δ(R2/R1), for each single-arginine mutant as compared with the WT sample highlight regions that are most affected by arginine charge neutralization (Figures 3C, D). While the hnNOE data is noisier due to the intrinsic lower sensitivity of the experiment, both difference plots display similar trends. An initial assessment of the overall difference between each mutant and WT can be made by summing the ΔhnNOE and Δ(R2/R1) across all H3 tail residues (Supplementary Table S3). A comparison of these sums ranks the overall effect of each individual arginine neutralization in the order of R26Q >> R8Q > R17Q > R2Q for ΔhnNOE or R8Q ≈ R17Q > R26Q >> R2Q for Δ(R2/R1). This sum, essentially the area between the WT and mutant profiles, captures both the magnitude of the effect on the dynamics of each tail residue and the breadth of the effect across the tail sequence. We sought to assess the breadth of the effect of each neutralization alone due to the important implications for tail accessibility and PTM crosstalk. The H3 tail region affected was defined by a string of at least three residues with non-overlapping error bars with interruption permitted by single residues with overlapping error bars (Table 1). By this definition, the breadth of effect ranks in the order of R26Q > R17Q = R8Q > R2Q for the hnNOE data and R26Q > R17Q > R8Q >> R2Q for the R2/R1 data. The breadth of effect is wider for the R2/R1 data, likely due in part to the lower sensitivity of the hnNOE. These rankings suggest that the R26Q individual mutation has the most extensive regional effect along the H3 tail sequence, consistent with its location adjacent to the putative ‘hinge’ region. As such, the neutralization of R26 extends the ‘hinge’ further N-terminally into the H3 tail. The smallest regional effect by R2Q is expected due to its position at the N-terminus, and the extent of effect is similar between R17Q and R8Q. For the most part, the effect is asymmetric, with a bias for the C-terminal side of the neutralized arginine position. The exception is for the R26Q R2/R1 data, which we speculate is due to: i) the broader effect observed for the R2/R1 data in general along with the tail endpoint at K36 creating a boundary condition, and ii) overall particle tumbling time starting to dominate near the core for R2/R1. While this asymmetric effect is simple to explain for R2Q, it is perhaps less obvious for the other three positions.

**TABLE 1:** Compilation of the breadth of the effect of arginine mutations on H3 tail dynamics within mutant-H3-NCP as compared to WT-H3-NCP. The H3 tail region affected by arginine neutralization was defined by a string of at least three residues with non-overlapping error bars (with possible interruption by single residues with overlapping error bars). The range was determined for each mutant from either hnNOE or R2/R1 data sets at 0 or 150 mM KCl. The boundaries of the region are listed along with the number of residues within this range for quick assessment of the effect. See hnNOE and R2/R1 data plotted in Figures 3, 5; Supplementary Figures S5, S7.

Mutant	hnNOE at 0 mM KCl	R2/R1 at 0 mM KCl	hnNOE at 150 mM KCl	R2/R1 at 150 mM KCl
R2Q	T3-Q5 (3 residue range)	T3-G12 (10 residue range)	A7-K9 (3 residue range)	T3-L20 (18 residue range)
R8Q	A7-A15 (9 residue range)	T3-T22 (20 residue range)	A7-A15 (9 residue range)	K4-R26 (23 residue range)
R17Q	K14-T22 (9 residue range)	R8-A29 (22 residue range)	A15-L20, A24-R26 (6 + 3 residue range)	T6-A31 (26 residue range)
R26Q	T22-V35 (14 residue range)	G13-V35 (23 residue range)	A24-V35 (12 residue range)	G13-V35 (23 residue range)
R2/8/17/26Q	T3-G34 (32 residue range)	T3-K36 (34 residue range)	T3-K36 (34 residue range)	T3-K36 (34 residue range)

Each of the single-arginine mutations affects the dynamics of at least one other H3 tail arginine residue. R2Q and R17Q influence the R8 position while R8Q and R26Q influence the R17 position. While the R2 position could not be measured directly, R8Q influences through to the T3 position, suggesting the potential of influencing R2. The R26 position is not significantly affected by individual neutralization of the three other arginine positions. Together, this speaks to crosstalk between arginine residues.

As already mentioned, the quadruple mutant has a global effect on the H3 tail. To gain insight into whether the effects of each arginine neutralization are additive or show indications of cooperative effects, the ΔhnNOE and Δ(R2/R1) were summed for all four single-arginine mutations and compared with the data for R2/8/17/26Q-H3-NCP (Figures 3C, D, black vs. red). The sum of the Δ(R2/R1) is remarkably similar to the Δ(R2/R1) for R2/8/17/26Q-H3-NCP. The sum of the ΔhnNOE suggests some potential variations as compared to the ΔhnNOE for R2/8/17/26Q-H3-NCP, but the hnNOE data is noisier than the R1 and R2 data. Thus, the data to this point largely support a model of additive effects.

### 3.5 Trends hold at physiological monovalent salt concentrations of 150 mM KCl

**FIGURE 4:**
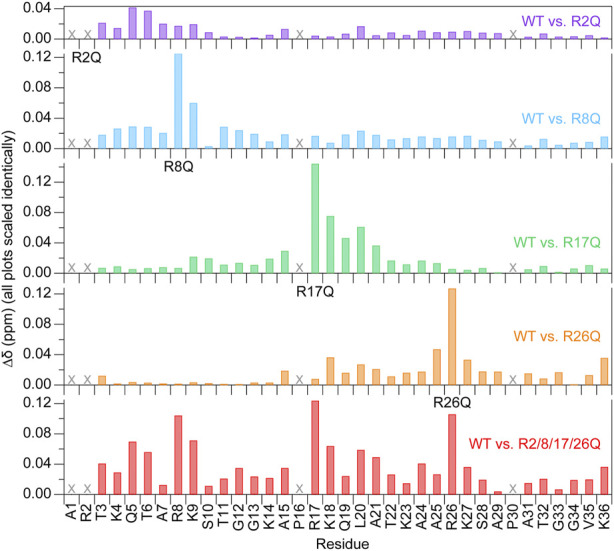
Chemical shift perturbations are localized near the mutated arginine at 150 mM KCl. Chemical shift perturbations ( ∆δ ) are plotted as a function of H3 tail residue for ^1^H-^15^N HSQC spectra of each mutant as compared to WT-H3-NCP at 150 mM KCl. “X” symbols mark residues that are not visible. Data were collected at 800 MHz and 304 K.

**FIGURE 5:**
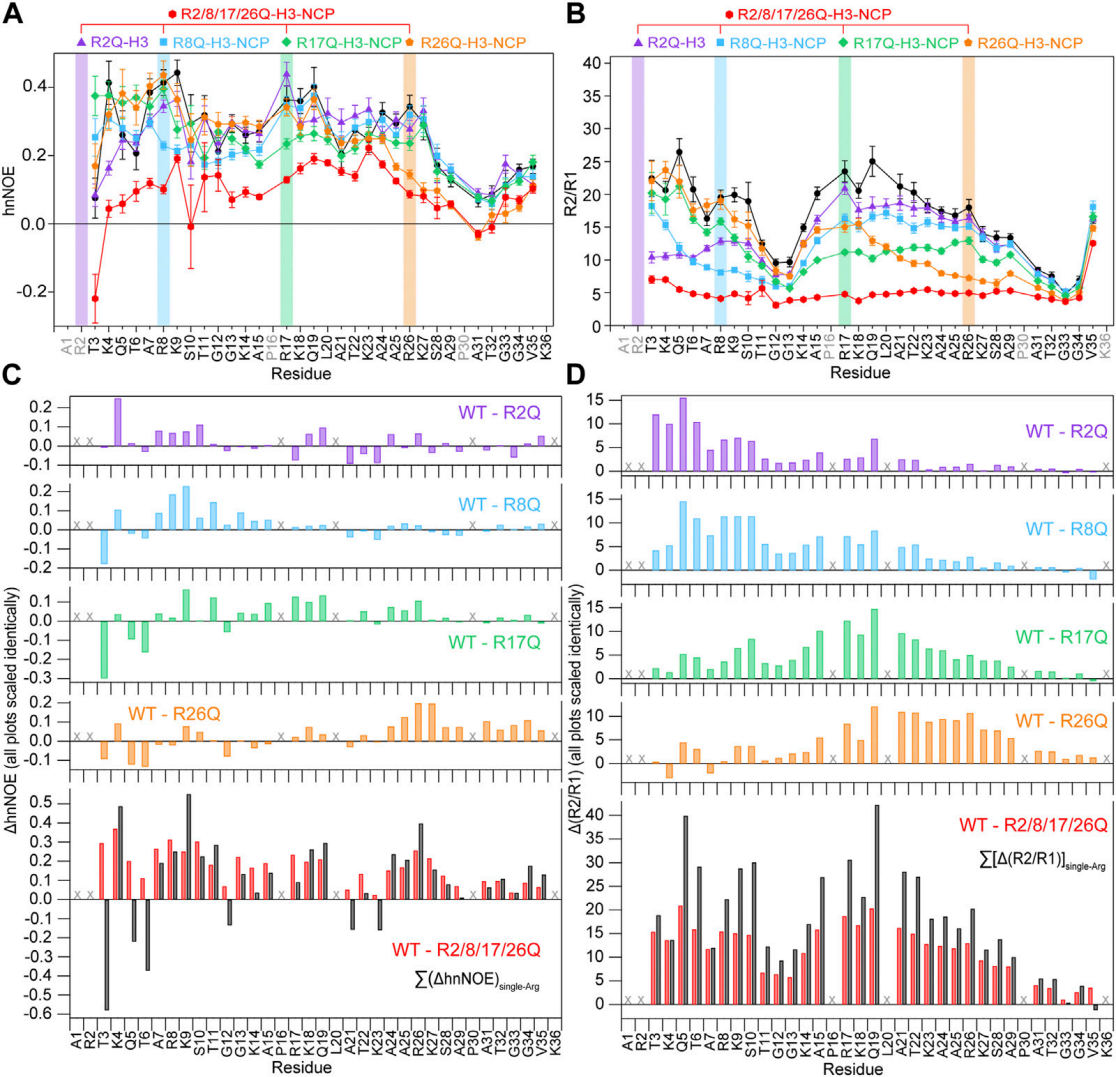
Neutralizing H3 tail arginines leads to a more global increase in H3 tail dynamics at 150 mM KCl. Plots are shown for **(A)** hnNOE values and **(B)** R2/R1 as a function of residue for WT- (black circles), R2Q- (purple triangles), R8Q- (blue squares), R17Q- (green diamonds), R26Q- (orange pentagons), and R2/8/17/26Q-H3-NCP (red hexagons). Error bars represent standard error propagation of the spectral noise for hnNOE values and were determined via the covariance matrix in fitting R1 and R2 decay curves, which was subsequently propagated for R2/R1. Note that some error bars are smaller than the symbols. Difference plots for **(C)** hnNOE and **(D)** R2/R1 show the difference between the per-residue hnNOE or R2/R1 for the H3 tail within ^15^N-WT-H3-NCP and within each single-arginine mutant (top four plots). The bottom plots show the difference between WT- and R2/8/17/26Q-H3-NCP (red) and the sum of the four difference plots for each single-arginine mutant (i.e., ΔhnNOE_WT-R2Q_ + ΔhnNOE_WT-R8Q_ + ΔhnNOE_WT-R17Q_ + ΔhnNOE_WT-R26Q_ on the lower left and Δ(R2/R1)_WT-R2Q_ + *Δ*(R2/R1)_WT-R8Q_ + *Δ*(R2/R1)_WT-R17Q_ + *Δ*(R2/R1)_WT-R26Q_ on the lower right) (black). “X” symbols mark residues omitted from analysis. Data were collected at 150 mM KCl, 800 MHz, and 304 K.

We next sought to investigate the effect of monovalent salt concentrations more representative of physiological conditions by conducting the same experiments at 150 mM KCl (Figures 4, 5; Supplementary Figures S3, S6, S7; Supplementary Table S2). Peaks observed as doublets at 0 mM KCl are observed as single peaks at 150 mM KCl, suggesting that salt lessens the effect of the NCP asymmetry on the H3 tail conformational ensemble. Spectral quality declines with the added salt, making analysis less robust, especially for the hnNOE. For all samples in the presence of 150 mM KCl, there is a global decrease in the hnNOE values, increase in R1, and decrease in R2 (along with a corresponding decrease in R2/R1 ratio) across the length of the H3 tail preceding the putative ‘hinge’ region as compared to 0 mM KCl (Figures 5A, B; Supplementary Figure S8). The ‘hinge’ region is retained at 150 mM KCl and also shows a decrease in the hnNOE but not R2/R1 as compared to 0 mM KCl, again likely due to differential contributions from overall tumbling. Together, these changes support an overall increase in ps-ns timescale dynamics with increasing monovalent salt as has been observed previously (Furukawa et al., 2020). The changes further support a model of the dynamics being dictated largely by electrostatically-driven tail interactions with DNA. However, the data do not exclude the possibility that the internal motions are also encoded by the H3 tail sequence itself.

The effect of KCl was compared across single-arginine mutants. Difference plots of the hnNOE and R2/R1 values show similar trends with the single-arginine mutants as were observed in the absence of KCl, supporting the relevance of the data collected at 0 mM KCl (Figures 3C, D; Figures 5C, D). Summing the ΔhnNOE and Δ(R2/R1) across all H3 tail residues ranks the overall effect of each individual arginine neutralization in the same or similar order as at 0 mM KCl: R26Q > R8Q > R17Q > R2Q for ΔhnNOE or R17Q > R8Q > R26Q > R2Q for Δ(R2/R1) (Supplementary Table S3). We conducted the same assessment of the breadth of the effect of each neutralization alone that was done at 0 mM KCl (Table 1). In terms of R2/R1, the affected range is larger for R2Q, R8Q, and R17Q at 150 mM KCl than 0 mM KCl, suggesting the possibility of a more extensive influence of each of these individual neutralizations across the H3 tail. The breadth of effect ranks in the order of R26Q > R17Q = R8Q > R2Q for the hnNOE data and R17Q > R26Q = R8Q > R2Q for the R2/R1 data. As expected, R2Q still has the smallest regional effect, but with a dramatic increase from 0 mM KCl as determined from R2/R1 (Table 1). Surprisingly, R26Q no longer has the broadest effect with respect to the R2/R1 data, which may be due to the same points discussed in Section 3.4.

As was observed at 0 mM KCl, the H3 tail within R2/8/17/26Q-H3-NCP shows a global increase in ps-ns timescale dynamics as compared to WT (Figures 5C, D; Table 1; Supplementary Figure S7). The quadruple mutant loses the dip in the R2/R1 profile at the first TGG (residues 11–13) at 150 mM KCl, which could be due to changes in overall tumbling contributions or hint at sequence-encoded internal motions in light of the preserved hnNOE profile shape. In contrast to 0 mM KCl conditions, the sum of the Δ(R2/R1) for all four single-arginine mutations is greater than the Δ(R2/R1) for R2/8/17/26Q-H3-NCP (Figure 5D, black vs. red) across the length of the H3 tail, supporting the broader effect of each single-arginine neutralization at 150 mM KCl.

## 4 Discussion

In this study, we begin a systematic characterization via NMR spin relaxation of modifications to individual H3 tail residues on the dynamic ensemble of conformations experienced by the H3 tail within the NCP. Here, we focus on the neutralization of arginine residues and demonstrate that arginines play a role in anchoring H3 tail dynamics. Mutation of all four arginine residues (R2, R8, R17, and R26) leads to a global increase in H3 tail mobility on the ps-ns timescale. Comparison of the single-arginine mutants to the extremes of WT- and R2/8/17/26Q-H3-NCP reveals that the hnNOE and R2/R1 profiles are depressed from WT levels toward R2/8/17/26Q levels regionally, up to ∼70% of the tail, rather than simply locally around the site of mutation. Monovalent salt concentrations of 150 mM KCl broaden the regional increase in ps-ns dynamics. The effect on slower timescale motions (µs-s) remains untested. Altogether, these studies support an emerging model wherein histone tail dynamics are an important part of the histone language.

**FIGURE 6:**
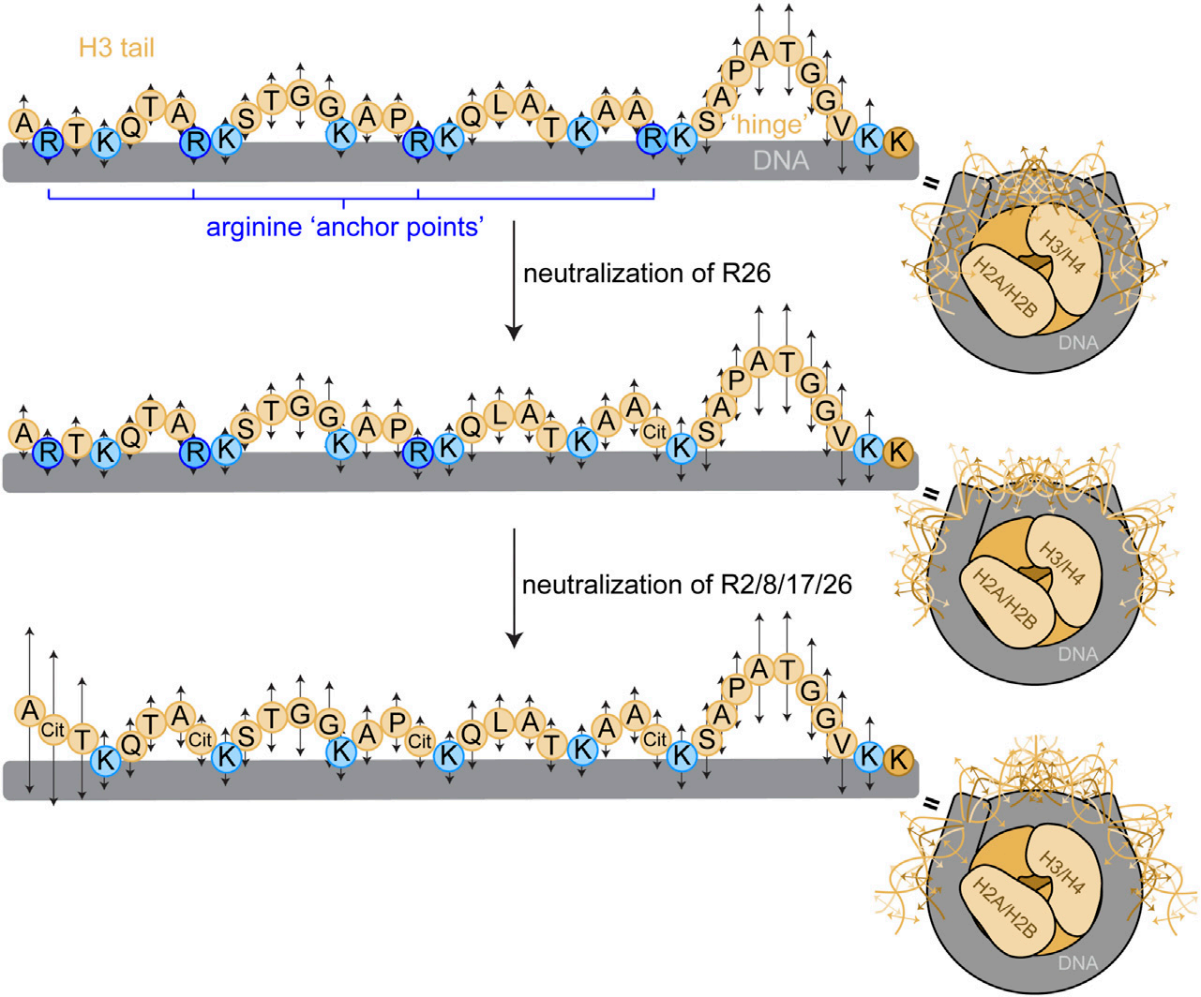
Proposed model for arginine ‘anchor points’ in H3 tail dynamics. We propose a model wherein H3 tail residues exist in a dynamic ensemble of DNA-bound conformations, and each residue has a microscopic site-binding constant for DNA that dictates the local, fast-timescale (ps-ns) dynamics of the residue (represented by arrows). Basic residues have stronger site-binding constants and thus slower motions (shorter arrows). Uncharged residues have a weaker site-binding constant and thus faster motions (longer arrows), and longer stretches of uncharged residues build up to faster dynamics as in the TGG repeats and the putative ‘hinge’ region. Mutating arginine anchor points increases the regional mobility around the arginine position by weakening the microscopic site-binding constant for DNA. These ps-ns timescale motions build up to slower timescale motions that involve global tail conformational rearrangements, illustrated by the conformational ensemble (only a few discrete states of a broad ensemble depicted) of the H3 tails within the NCP (right). Sequential neutralization of charges would have a profound effect on tail accessibility. A cartoon depiction of the NCP shows a complex of two H3/H4 dimers and two H2A/H2B dimers (shades of beige) wrapped by DNA (grey) as in Figure 1. The H3 tail sequence is explicitly depicted with circles representing residues. The bulk of the tail is represented in light beige with basic residues highlighted in blue, arginine in dark blue and lysine in light blue.

H3 tail mobility is constrained, but not rigid, within the NCP; the H3 tails exist in a dynamic ensemble of DNA-bound conformations within the NCP (Stützer et al., 2016; Morrison et al., 2018; Lehmann et al., 2020; Ghoneim et al., 2021; Morrison et al., 2021; Zandian et al., 2021; Tsunaka et al., 2022). We propose a model wherein arginines are ‘anchor points’ that are distributed relatively evenly along the length of the H3 tail (Figure 6). While overall, or macroscopically, the tail exists in DNA-bound conformations, locally, the tail fluctuates between bound and released in a manner governed by microscopic binding constants (and corresponding on/off rates) for each residue. These complexes comprised of large ensembles of states are also referred to as “fuzzy” complexes (Tompa and Fuxreiter, 2008; Dyson, 2012; Borgia et al., 2018; Fuxreiter, 2018; Ghoneim et al., 2021). Basic residues (arginine and lysine) are expected to have the tightest microscopic binding affinities, which would influence neighboring residues. Neutralization of arginine residues weakens the microscopic binding constant at that position, increasing its dynamics. In this study, we show that neutralizing a single arginine residue increases the regional dynamics of the tail in a position-dependent manner. R26Q and R2Q affect the widest and narrowest swath of residues, respectively. Interestingly, R26 and R8 displayed enhanced interactions with bases within the minor groove in molecular dynamics simulations (Shaytan et al., 2016). Remarkably, neutralization of R8, R17, or R26 at 150 mM KCl increases the ps-ns dynamics of ∼60–70% of the tail, and even R2 increases the dynamics of ∼50% of the tail. A flexible ‘hinge’ region (Furukawa et al., 2020; Morrison et al., 2021; Tsunaka et al., 2022) exists in residues S28-K36, and K36 is the first H3 tail residue visible where the tail emerges from between the two gyres of DNA. In our model, this ‘hinge’ is pinned by R26, and neutralizing this anchor point expands the ‘hinge’ further N-terminally into the tail. We speculate that R26 could serve as a key anchor point whose neutralization serves to unzip the tail and expand accessibility to readers and other regulatory proteins/complexes starting from the ‘hinge’ and moving towards the N-terminus. In this model, the ps-ns timescale dynamics build up to slower timescale dynamics that involve global tail conformational rearrangements, which remains to be tested experimentally.

The data reported here are interpreted as reporting on ps-ns timescale dynamics of the H3 tail. The effective CPMG field strength used in the R2 experiments (500 Hz) may not fully suppress any µs-ms timescale dynamics within the H3 tail. Thus, the reported R2 rates may have contributions from µs-ms timescale motions, which could also be differentially affected by the arginine mutations. Future experiments will be required to measure dynamics across a wider range of timescales. In addition, the experiments reported in this study were only conducted at a single concentration of NCP. We interpret the data as though we are only monitoring histone tail dynamics from intra-nucleosomal interactions with DNA. However, we cannot distinguish between intra- and inter-nucleosomal tail-DNA interactions at this point. Using salt-dependent data from nucleosome arrays (Zheng et al., 2005), we speculate that at 0 mM KCl, interactions are predominantly intra-nucleosomal while at 150 mM KCl, dynamic ensembles of intra- and inter-nucleosomal interactions exist. Particles with only intra- or a combination of intra- and inter-nucleosomal interactions would have different effective rotational correlation times and thus differentially affect R2 and R1 relaxation. In addition, mutations at different positions have the potential to shift populations of intra- and inter-nucleosomal interactions. These are currently experimental limitations.

We suggest that the neutralization of arginine via mutation to glutamine provides insight into citrullination, which is the deimination of arginine carried out by protein arginine deiminases (PADs) (Fuhrmann et al., 2015; Mondal and Thompson, 2019). Citrullination is a particularly intriguing candidate for a direct functional role of regulation by histone tail dynamics. The mechanism by which citrullination of histones regulates chromatin is not well understood, in part because citrullination does not have a plethora of known readers and has no known erasers (Fuhrmann et al., 2015). In fact, only one reader of citrullination has been identified thus far (Xiao et al., 2017). H3 tail targets for PAD enzymes have been identified, underlining the importance of studying the effect of neutralizing H3 tail arginines. For example, H3R26 is citrullinated by PAD2 at estrogen receptor *α* target genes, which is associated with a local opening of chromatin and activation of gene expression (Zhang et al., 2012; Clancy et al., 2017). In addition, PAD4 citrullination of H3 is associated with neutrophil extracellular trap formation (NETosis), which is accompanied by global DNA decompaction (Lewis et al., 2015; Kenny et al., 2017). It is a compelling model to consider the possibility of citrullination functioning through a direct effect on chromatin interactions and dynamics. This model requires further investigation.

In this study, we only perturb H3 tail arginine residues. All the lysines remain, spaced relatively evenly along the length of the tail as potential multivalent interaction points with DNA (Figure 6). In general, arginine and to a lesser extent lysine dominate interactions with DNA (Rohs et al., 2009). Within the NCP, histone tail arginines largely have access to a widened narrow groove as opposed to the preferred narrowed minor groove. This is in contrast to the nucleosome, which includes linker DNA and the potential for a narrower minor groove. Within the nucleosome, several simulation studies have indicated more contacts formed by individual H3 tail arginines than lysines with DNA (Li and Kono, 2016; Peng et al., 2021). Other simulations suggest that even within the NCP, H3 tail arginines have more energetically-favorable interactions with DNA than lysines (Morrison et al., 2018), which may be related to the more favorable enthalpy and number of interactions between arginines and phosphates (Mascotti and Lohman, 1997; DeRouchey et al., 2013). Similar observations have been made with the H4 tail within NCP where simulations captured more DNA interactions with arginine than lysine, and NMR ^15^N-R2 relaxation experiments revealed an important arginine anchor point in H4 R3 (Rabdano et al., 2021). Other MD simulations indicate that overall, even within the NCP, histone tail arginines and lysines have similar numbers of DNA interactions but with a unique preference of arginines for minor groove interactions (Armeev et al., 2021). Ongoing investigations will provide additional insight into the relative contributions of H3 tail arginine and lysine residues to tail dynamics.

In recent years, a model has been emerging wherein histone tail dynamics are an important part of the histone language. A variety of nuclear factors can perturb the ensemble of DNA-bound histone tail conformations. We previously demonstrated that the conformational ensemble of the H3 tails is sensitive to the assembly state of the NCP, with the H3 tails more dynamic within tetrasome than within NCP (Morrison et al., 2021). In addition, partial replacement of NCP DNA by the pAID of FACT leads to an increase in ps-ns dynamics of the H3 tail (Tsunaka et al., 2020). Rotational correlation times calculated from ^15^N-R1 and R2 data show reduced ns-timescale dynamics for the H3 tails within nucleosome as compared to NCP, which is further reduced upon the binding of linker histone H1 (Stützer et al., 2016; Zandian et al., 2021), but these values may be convoluted by changes in the overall rotational correlation time of the complex. A different study indicated increased ps-ns timescale dynamics for the H3 tails within nucleosome as compared to NCP (Furukawa et al., 2022). Charge-modulating histone PTMs also contribute to changes in histone tail dynamics. To date, this has been observed for the H3 tail within nucleosomes acetylated by Gcn5 (includes H3 K14ac) and phosphorylated by Aurora B (includes H3 S10ph and S28ph) (Stützer et al., 2016). These modifications globally increased the ns-timescale dynamics of the H3 tails outside of the ‘hinge’ region, as reported by calculated rotational correlation times (Stützer et al., 2016). Interestingly, incorporating a phosphorylation-mimetic at only a single H3 tail position, S28, within the nucleosome resulted in a global reduction in the rotational correlation time across the tail (Pelaz et al., 2020). This suggests the possibility of a stronger effect of adding a single negative charge (via H3 S28E) than neutralizing a single positive charge (via the single-arginine mutations reported in this paper). We speculate that the strong effect of H3 S28E could also be related to its position within the ‘hinge’ region. Our systematic investigation of arginine neutralization with implications for citrullination adds to this growing body of work. This study along with models for histone tail dynamics in the histone language have implications for crosstalk between histone PTMs (Fischle, 2008; Lee et al., 2010; Winter and Fischle, 2010; Stützer et al., 2016; Morrison et al., 2018; Peng et al., 2021). The regional rather than simply local influence of neutralization of each arginine, especially R26, means an increase in dynamics for other heavily modified H3 tail positions including K4, K9, S10, K27, and S28. In addition, neutralization of a single H3 tail arginine increases the dynamics of at least one other H3 tail arginine. The increase in ps-ns motions is presumably accompanied by an increase in accessibility of these residues to modification by writer complexes. Sequential neutralizations of arginine and lysine and phosphorylation of serine would have profound effects on tail accessibility and interactions. Future studies will provide further insight into the role of histone tail dynamics in the histone language and its importance in PTM crosstalk.

## Data Availability

The relaxation datasets presented in this study have been deposited in the Biological Magnetic Resonance Data Bank (BMRB). The six BRMB deposition numbers for the paper are as follows: 51930, 51931, 51932, 51933, 51934, 51935. Datasets are available on request. The raw data supporting the conclusions of this article will be made available by the authors, without undue reservation.
