# Supplementary material for: Arginine anchor points govern H3 tail dynamics
Source: Front Mol Biosci. 2023 May 2;10:1150400. doi: 10.3389/fmolb.2023.1150400 (PMC10228543; doi:10.3389/fmolb.2023.1150400)
Supplement: Supplementary file 1 [file DataSheet1.pdf]

Supplementary Material

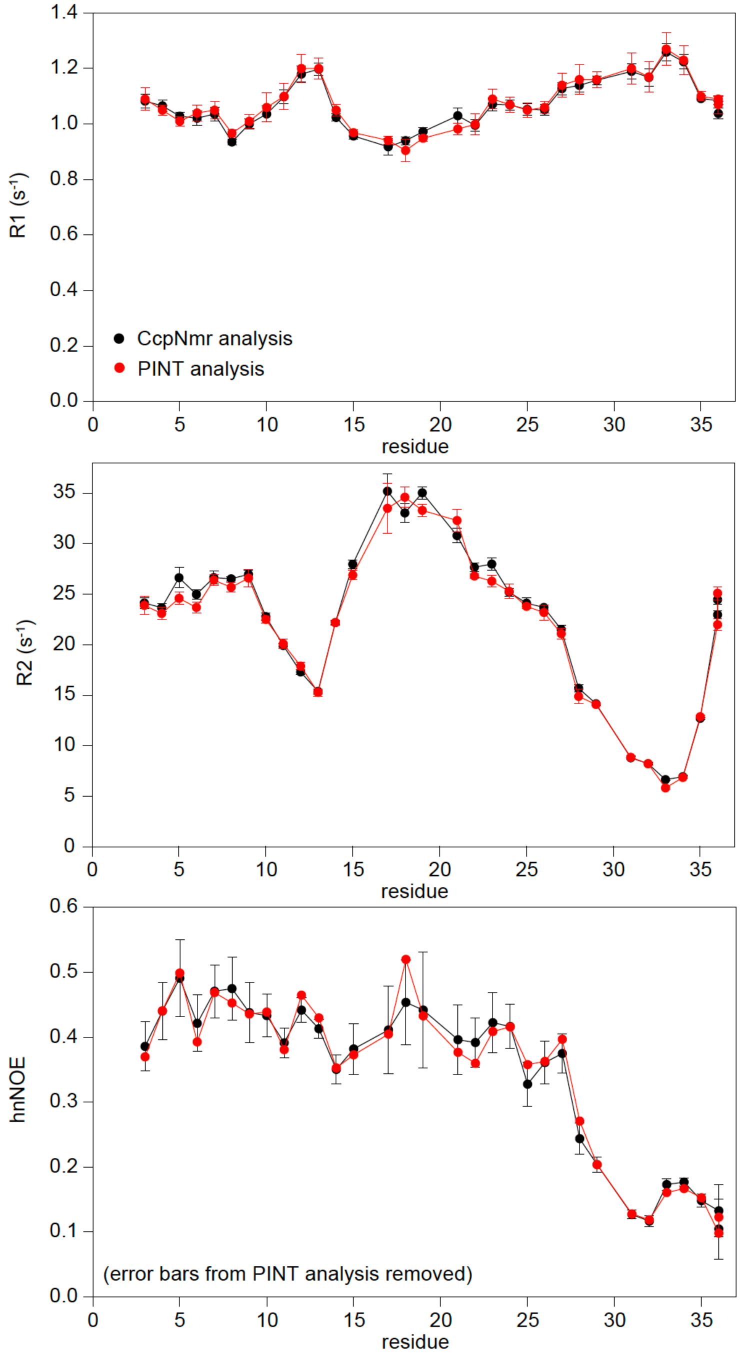

Supplementary Figure S1. Comparison of R1, R2, and hnNOE values for data collected on ^{15}N-WT-H3-NCP at 0 mM KCl and analyzed using either CcpNmr Analysis (black) or PINT (red). For analysis in CcpNmr, error bars represent standard error propagation of the spectral noise for hnNOE values and were determined via the covariance matrix in fitting R1 and R2 decay curves. For analysis in PINT, error bars for R1 and R2 were determined via jackknife analysis. For clarity, error bars were omitted from the plot for hnNOE analysis in PINT because PINT-derived errors ranged from 0.7-1.8. Similar results from CcpNmr Analysis and PINT support a robust analysis.

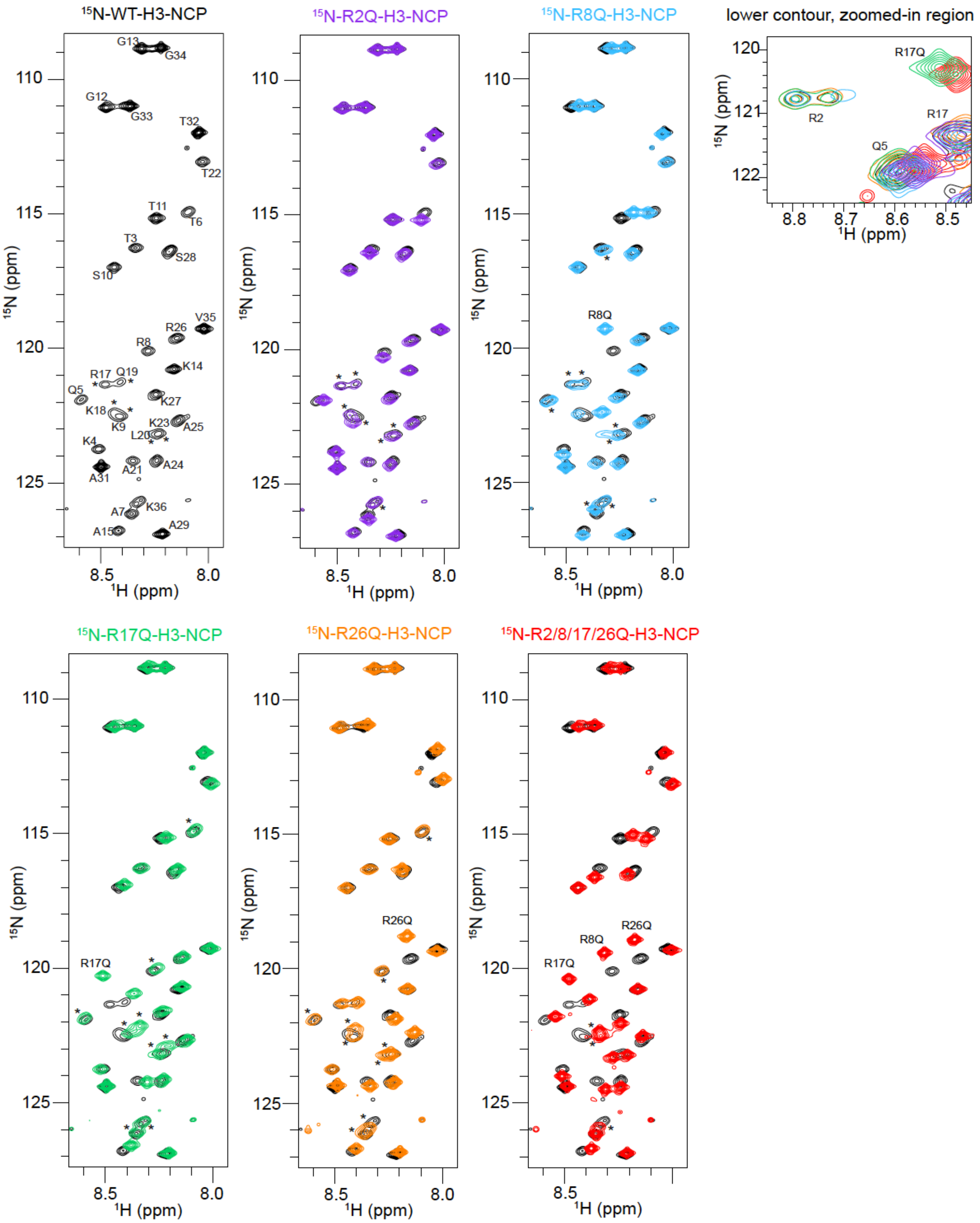

Supplementary Figure S2. Overlays of ^{1}H-^{15}N HSQC spectra of ^{15}N-WT-H3-NCP with each ^{15}N-mutant-H3-NCP at 0 mM KCl. Spectra are color-coded according to the labels and displayed at the same contour. Assignments are labeled in the WT-H3-NCP spectrum, and arginine-to-glutamine mutations are labeled in overlays. Residues with peak overlap are denoted (*). An additional overlay (top right) of all six spectra is displayed at a lower contour to show residue R2. Data were collected at 800 MHz and 304 K.

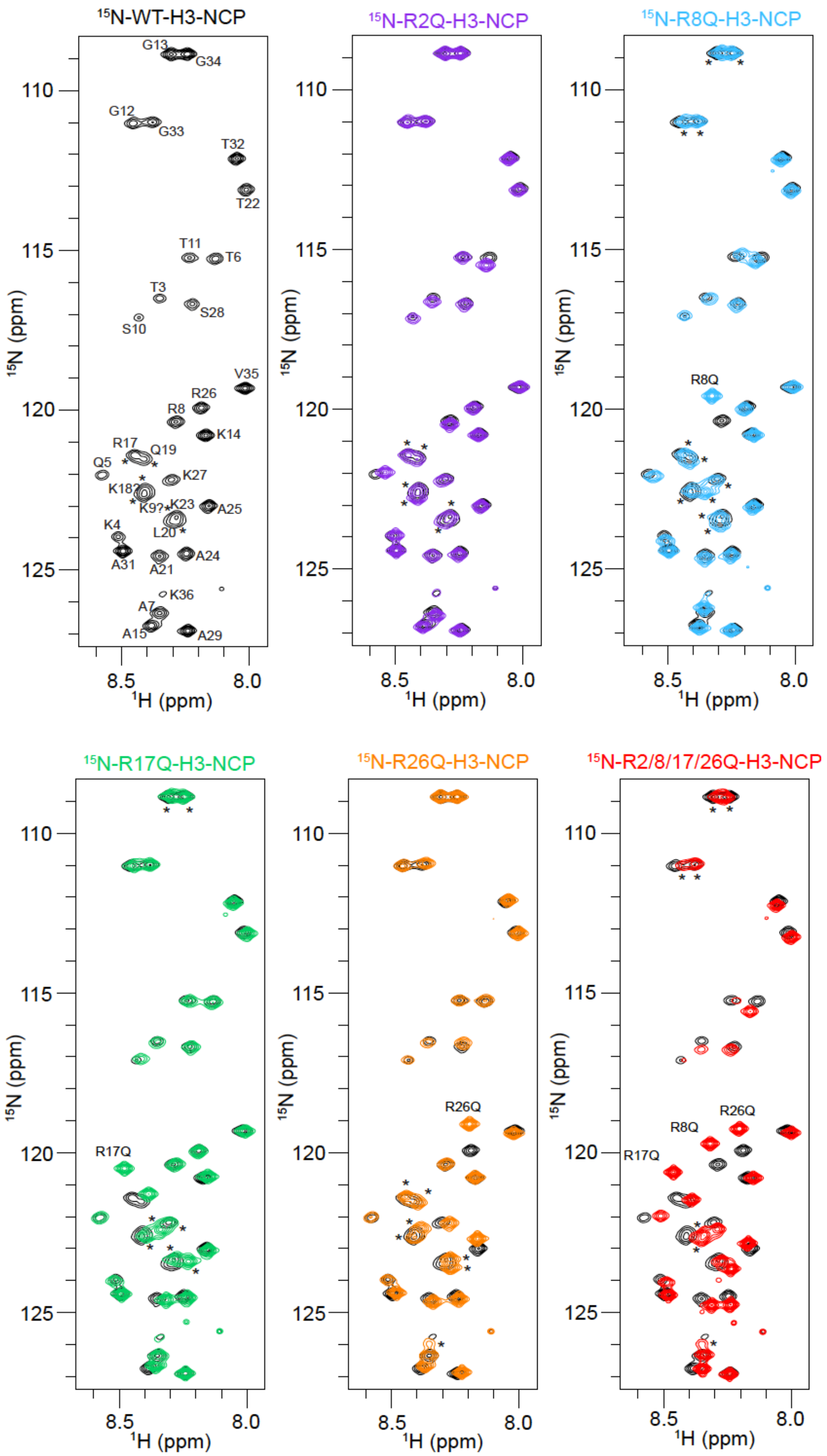

Supplementary Figure S3. Overlays of ^{1}H-^{15}N HSQC spectra of ^{15}N-WT-H3-NCP with each ^{15}N-mutant-H3-NCP at 150 mM KCl. Spectra are color-coded according to the labels. Assignments are labeled in the WT-H3-NCP spectrum, and arginine-to-glutamine mutations are labeled in overlays. Residues with peak overlap are denoted (*). Data were collected at 800 MHz and 304 K.

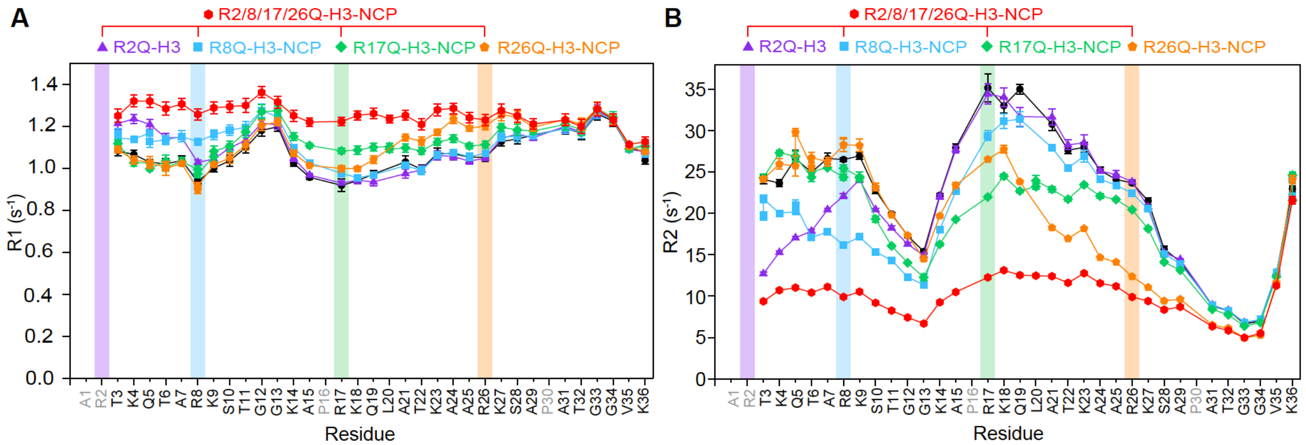

Supplementary Figure S4. Plot of (**A**) ^{15}N-R1 rates and (**B**) ^{15}N-R2 rates as a function of residue for WT- (black circles), R2Q- (purple triangles), R8Q- (blue squares), R17Q- (green diamonds), R26Q- (orange pentagons), and R2/8/17/26Q-H3-NCP (red hexagons) at 0 mM KCl. Error bars were determined via the covariance matrix in fitting R1 and R2 decay curves. Note that some error bars are smaller than the symbols. Residues without data for any sample are colored grey in the x-axis labels. Data were collected at 800 MHz and 304 K.

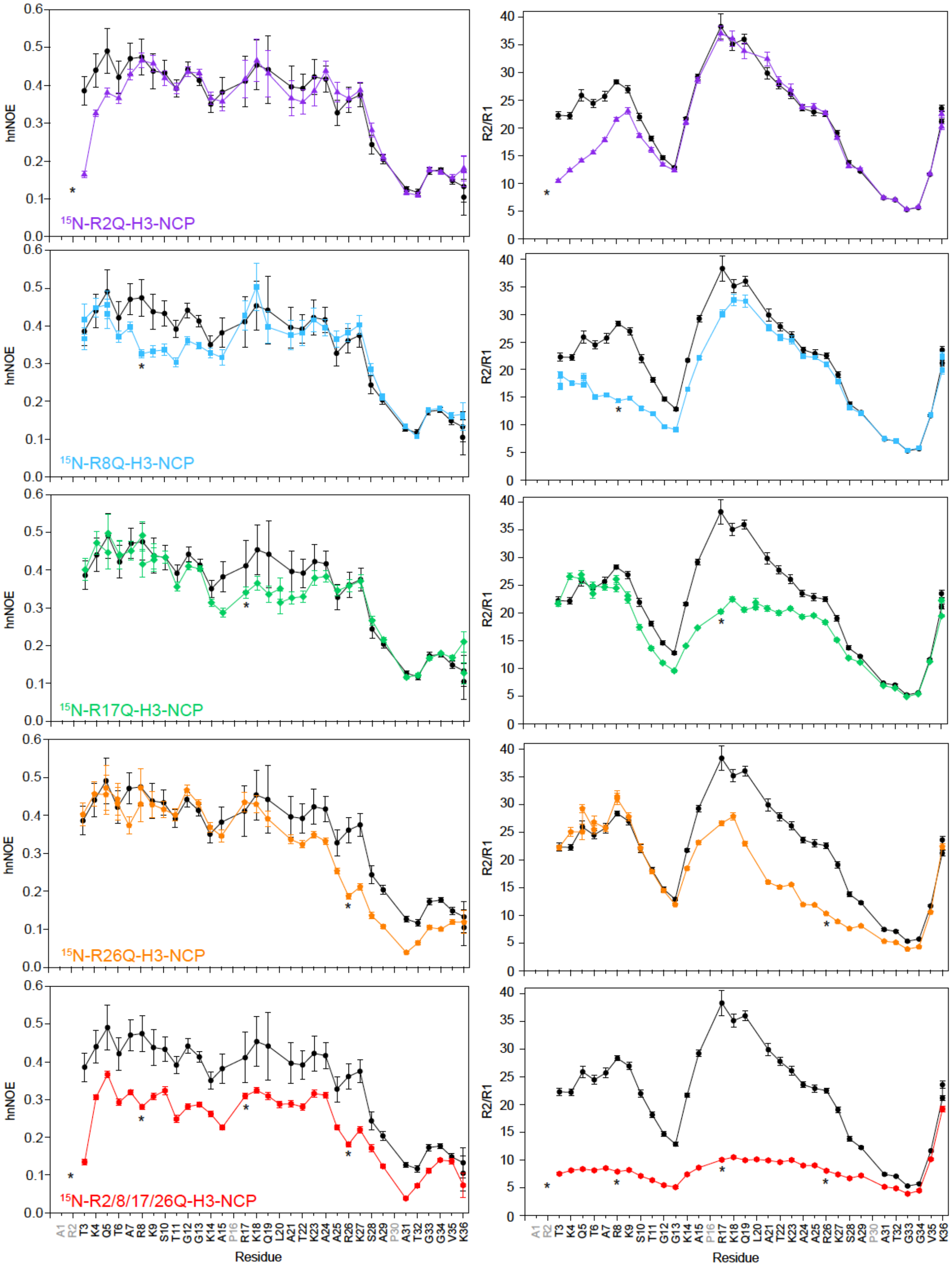

Supplementary Figure S5. Amide nuclear spin relaxation data is shown for each ^{15}N-mutant-H3-NCP plotted with WT (black circles) for comparison in 0 mM KCl conditions. Plots of hnNOE values (left) and R2/R1 (right) as a function of H3 tail residue for R2Q- (purple triangles), R8Q- (blue squares), R17Q- (green diamonds), R26Q- (orange pentagons), and R2/8/17/26Q-H3-NCP (red hexagons). Mutated positions are marked by an asterisk. Error bars represent standard error propagation of the spectral noise for hnNOE values and were determined via the covariance matrix in fitting R1 and R2 decay curves, which was subsequently propagated for R2/R1. Note that some error

bars are smaller than the symbols. Residues without data for any sample are colored grey in the x-axis labels. Data were collected at 800 MHz and 304 K.

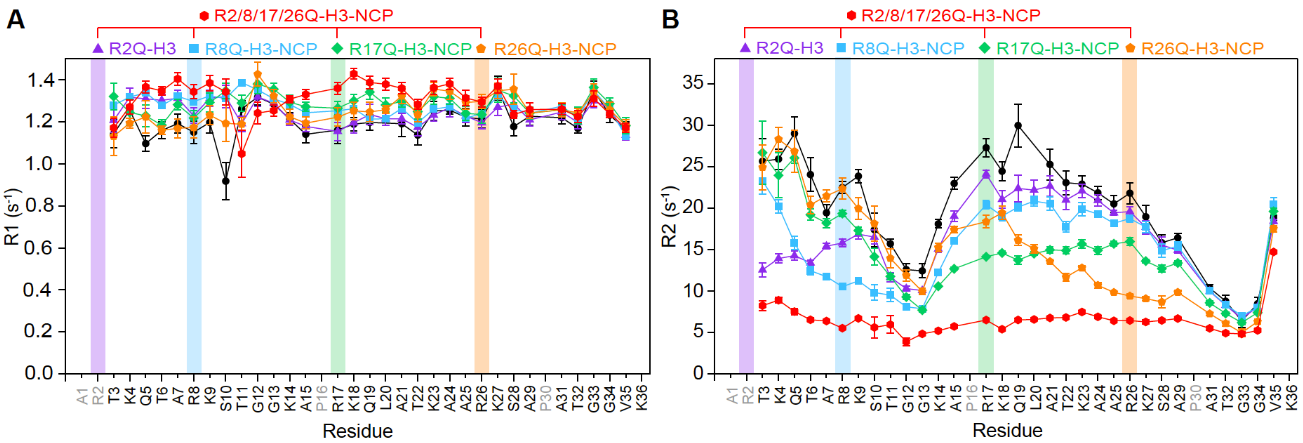

Supplementary Figure S6. Plot of (**A**) ^{15}N-R1 rates and (**B**) ^{15}N-R2 rates as a function of residue for WT- (black circles), R2Q- (purple triangles), R8Q- (blue squares), R17Q- (green diamonds), R26Q- (orange pentagons), and R2/8/17/26Q-H3-NCP (red hexagons) at 150 mM KCl. Error bars were determined via the covariance matrix in fitting R1 and R2 decay curves. Note that some error bars are smaller than the symbols. Residues without data for any sample are colored grey in the x-axis labels. Data were collected at 800 MHz and 304 K.

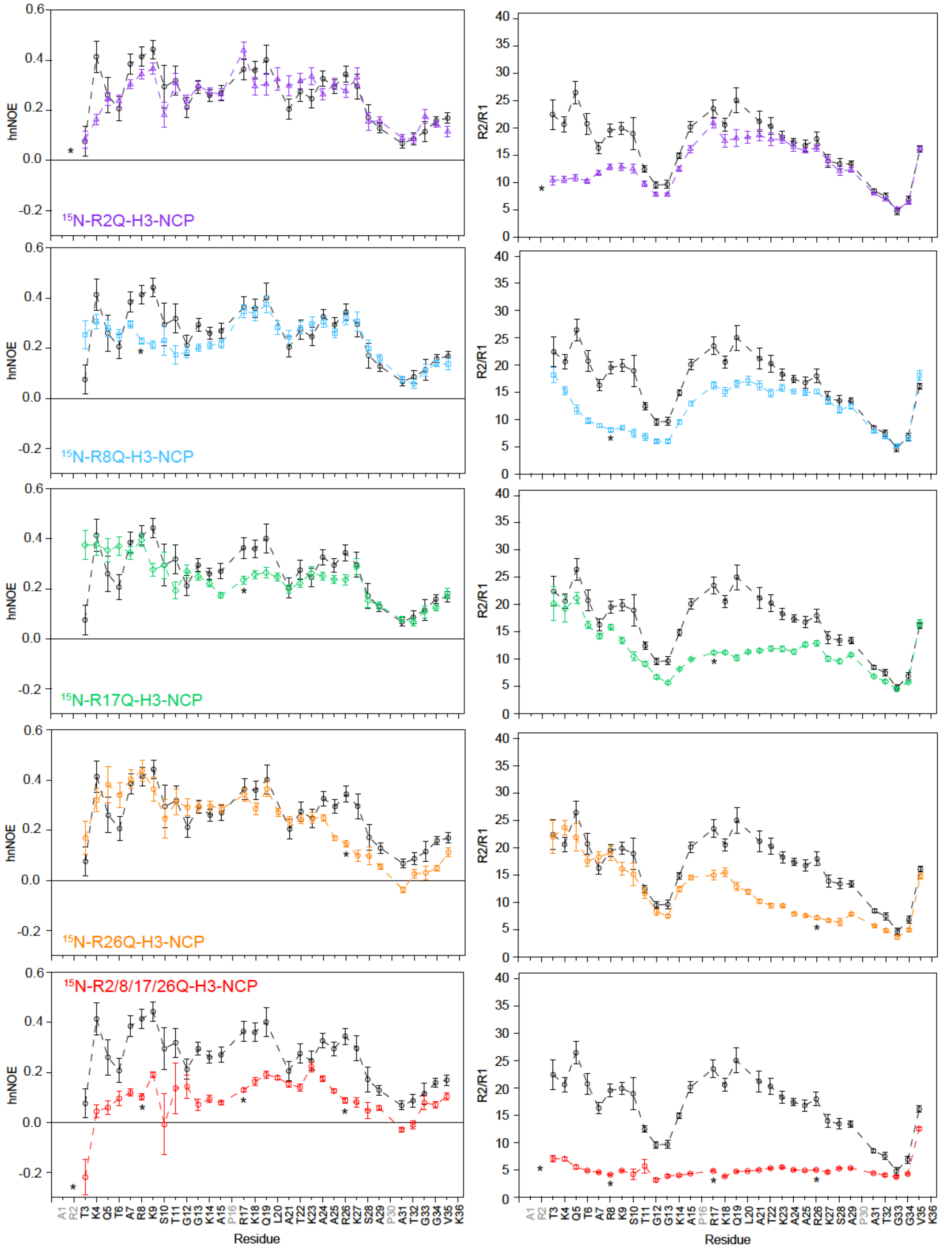

Supplementary Figure S7. Amide nuclear spin relaxation data is shown for each ^{15}N-mutant-H3-NCP plotted with WT (black circles) for comparison in 150 mM KCl conditions. Plots of hnNOE values (left) and R2/R1 (right) as a function of H3 tail residue for R2Q- (purple triangles), R8Q- (blue squares), R17Q- (green diamonds), R26Q- (orange pentagons), and R2/8/17/26Q-H3-NCP (red hexagons). Mutated positions are marked by an asterisk. Error bars represent standard error propagation of the spectral noise for hnNOE values and were determined via the covariance matrix in fitting R1 and R2 decay curves, which was subsequently propagated for R2/R1. Note that some error

bars are smaller than the symbols. Residues without data for any sample are colored grey in the x-axis labels. Data were collected at 800 MHz and 304 K.

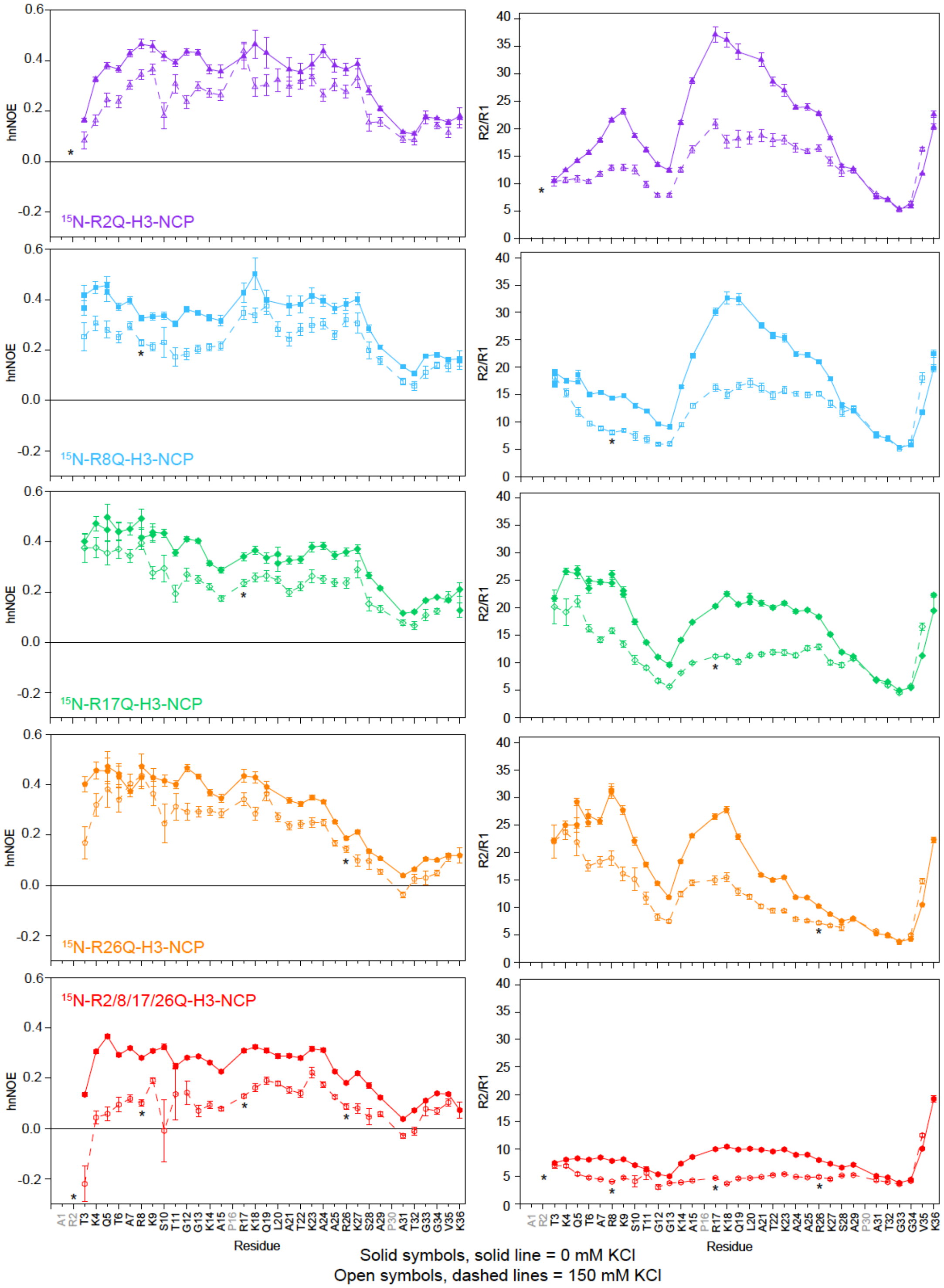

Supplementary Figure S8. Amide nuclear spin relaxation data is shown for ^{15}N-mutant-H3-NCP in the absence (closed symbols, solid line) and presence (open symbols, dashed line) of 150 mM KCl. Plots of hnNOE values (left) and R2/R1 (right) as a function of H3 tail residue for R2Q- (purple triangles), R8Q- (blue squares), R17Q- (green diamonds), R26Q- (orange pentagons), and R2/8/17/26Q-H3-NCP (red hexagons). Error bars represent standard error propagation of the spectral noise for hnNOE values and were determined via the covariance matrix in fitting R1 and R2 decay

curves, which was subsequently propagated for R2/R1. Note that some error bars are smaller than the symbols. Residues without data for any sample are colored grey in the x-axis labels. Data were collected at 800 MHz and 304 K.

Supplementary Table S1. Compilation of average (including standard deviation), minimum, and maximum values for hnNOE, R2/R1, R1, and R2 for each WT or mutant ^{15}N-H3-NCP at 0 mM KCl. Values are listed for all analyzed residues in the H3 tail and separately for residues T3-K27 and S28-K36 (the 'hinge' region). See Materials and Methods for additional details and residues excluded from analysis.

hnNOE												
	WT		R2Q		R8Q		R17Q		R26Q		R2/8/17/26Q	
min(all)	0.10		0.11		0.11		0.12		0.04		0.04	
average(all)	0.34±	0.12	0.33±	0.11	0.33±	0.10	0.34±	0.11	0.32±	0.14	0.24±	0.09
max(all)	0.49		0.47		0.50		0.50		0.47		0.37	
min(3-27)	0.33		0.16		0.30		0.29		0.19		0.13	
average(3-27)	0.41±	0.04	0.39±	0.06	0.38±	0.05	0.39±	0.06	0.39±	0.08	0.28±	0.05
max(3-27)	0.49		0.47		0.50		0.50		0.47		0.37	
min(28-36)	0.10		0.11		0.11		0.12		0.04		0.04	
average(28-36)	0.16±	0.05	0.18±	0.05	0.18±	0.05	0.17±	0.05	0.10±	0.03	0.11±	0.04
max(28-36)	0.24		0.28		0.29		0.27		0.14		0.17	
**R2/R1 ratio**												
	WT		R2Q		R8Q		R17Q		R26Q		R2/8/17/26Q	
min(all)	5		5		5		5		4		4	
average(all)	21±	9	19±	9	17±	7	18±	6	18±	9	8±	3
max(all)	38		37		33		27		31		19	
min(3-27)	13		10		9		10		9		5	
average(3-27)	25 ±	6	22±	8	20±	7	21±	5	21±	7	8±	1
max(3-27)	38		37		33		27		31		10	
min(28-36)	5		5		5		5		4		4	
average(28-36)	12±	7	12±	6	12±	6	11 ±	6	8 ±	6	8±	5
max(28-36)	24		23		22		22		22		19	
**R1 (s^{-1})**												
	WT		R2Q		R8Q		R17Q		R26Q		R2/8/17/26Q	

min(all)	0.92		0.93		0.95		0.97		0.90		1.11	
average(all)	1.07±	0.09	1.10±	0.10	1.12±	0.08	1.11±	0.08	1.11±	0.10	1.25±	0.05
max(all)	1.26		1.27		1.27		1.28		1.29		1.36	
min(3-27)	0.92		0.93		0.95		0.97		0.90		1.21	
average(3-27)	1.04±	0.07	1.08±	0.10	1.11±	0.09	1.09±	0.07	1.08±	0.10	1.27±	0.04
max(3-27)	1.20		1.24		1.27		1.27		1.25		1.36	
min(28-36)	1.04		1.08		1.07		1.09		1.08		1.11	
average(28-36)	1.15±	0.07	1.16±	0.07	1.16±	0.06	1.18±	0.07	1.20±	0.07	1.21±	0.06
max(28-36)	1.26		1.27		1.26		1.28		1.29		1.28	

R2 (s^{-1})												
	WT		R2Q		R8Q		R17Q		R26Q		R2/8/17/26Q	
min(all)	7		7		7		6		5		5	
average(all)	22 ±	8	20 ±	8	19 ±	7	20±	6	19 ±	8	10 ±	3
max(all)	35		34		31		27		30		22	
min(3-27)	15		13		11		12		11		7	
average(3-27)	26±	5	23 ±	6	21 ±	6	22 ±	4	22±	6	11±	2
max(3-27)	35		34		31		27		30		13	
min(28-36)	7		7		7		6		5		5	
average(28-36)	13 ±	7	13 ±	6	13 ±	6	13±	7	10±	6	9 ±	5
max(28-36)	24		25		24		25		24		22	

Supplementary Table S2. Compilation of average (including standard deviation), minimum, and maximum values for hnNOE, R2/R1, R1, and R2 for each WT or mutant ^{15}N-H3-NCP at 150 mM KCl. Values are listed for all analyzed residues in the H3 tail and separately for residues T3-K27 and S28-K36 (the 'hinge' region). See Materials and Methods for additional details and residues excluded from analysis.

hnNOE												
	WT		R2Q		R8Q		R17Q		R26Q		R2/8/17/26Q	
min(all)	0.07		0.08		0.06		0.07		-0.04		-0.22	
average(all)	0.26±	0.11	0.25±	0.09	0.24±	0.08	0.24±	0.09	0.23±	0.13	0.09±	0.08
max(all)	0.44		0.44		0.38		0.40		0.44		0.22	
min(3-27)	0.08		0.08		0.17		0.17		0.10		-0.22	

average(3-27)	0.30±	0.08	0.28±	0.07	0.27±	0.05	0.27±	0.06	0.28±	0.08	0.11±	0.09
max(3-27)	0.44		0.44		0.38		0.40		0.44		0.22	
min(28-36)	0.07		0.09		0.06		0.07		-0.04		-0.03	
average(28-36)	0.13±	0.04	0.13±	0.04	0.13±	0.05	0.12±	0.04	0.05±	0.05	0.05±	0.05
max(28-36)	0.17		0.18		0.20		0.18		0.11		0.10	
R2/R1 ratio												
	WT		R2Q		R8Q		R17Q		R26Q		R2/8/17/26Q	
min(all)	5		5		5		5		4		3	
average(all)	17±	6	13 ±	4	12±	4	12±	4	12±	6	5 ±	2
max(all)	26		21		18		21		24		13	
min(3-27)	10		8		6		6		7		3	
average(3-27)	19±	4	14 ±	4	13±	4	12 ±	4	13 ±	5	5±	1
max(3-27)	26		21		18		21		24		7	
min(28-36)	5		5		5		5		4		4	
average(28-36)	10 ±	4	10±	4	10±	5	9±	4	7±	4	6±	3
max(28-36)	16		16		18		17		15		13	
R1 (s^{-1})												
	WT		R2Q		R8Q		R17Q		R26Q		R2/8/17/26Q	
min(all)	0.92		1.13		1.13		1.18		1.13		1.05	
average(all)	1.20±	0.08	1.24±	0.05	1.27±	0.05	1.28±	0.05	1.25±	0.07	1.30±	0.08
max(all)	1.36		1.32		1.39		1.38		1.43		1.43	
min(3-27)	0.92		1.15		1.20		1.18		1.13		1.05	
average(3-27)	1.19±	0.09	1.24±	0.05	1.28±	0.05	1.29±	0.05	1.25±	0.07	1.32±	0.08
max(3-27)	1.36		1.32		1.39		1.38		1.43		1.43	
min(28-36)	1.16		1.13		1.13		1.18		1.18		1.17	
average(28-36)	1.22±	0.06	1.23±	0.06	1.25±	0.07	1.27±	0.06	1.27±	0.06	1.24±	0.04
max(28-36)	1.35		1.30		1.35		1.36		1.35		1.31	
R2 (s^{-1})												
	WT		R2Q		R8Q		R17Q		R26Q		R2/8/17/26Q	
min(all)	6		7		7		6		5		4	
average(all)	20±	6	16 ±	5	15 ±	5	15±	5	15±	6	6 ±	2

max(all)	30	24	23	27	28	15
min(3-27)	12	10	8	8	9	4
average(3-27)	22± 5	17 ± 4	16± 5	16± 5	17± 6	6 ± 1
max(3-27)	30	24	23	27	28	9
min(28-36)	6	7	7	6	5	5
average(28-36)	12 ± 5	12± 5	12 ± 5	11± 5	9± 4	7± 4
max(28-36)	19	18	20	20	18	15

Supplementary Table S3. Compilation of average, standard deviation, and sum of all values for Δ(R2/R1) and ΔhnNOE for each mutant ^{15}N-H3-NCP as compared to WT at 0 or 150 mM KCl as specified. See Materials and Methods for additional details and residues excluded from analysis.

Data set	Parameter	R2Q	R8Q	R17Q	R26Q	R2/8/17/26Q
Δ(R2/R1) at 0 mM KCl	average (mean)	2	4	4	4	13
	standard deviation	4	4	5	6	8
	sum	69	138	137	128	409
Δ(R2/R1) at 150 mM KCl	average (mean)	4	5	5	5	12
	standard deviation	4	4	4	4	5
	Sum	110	146	152	138	347
ΔhnNOE at 0 mM KCl	average (mean)	0.01	0.02	0.02	0.04	0.12
	standard deviation	0.05	0.05	0.04	0.05	0.05
	sum	0.40	0.66	0.56	1.41	3.58
ΔhnNOE at 150 mM KCl	average (mean)	0.02	0.03	0.02	0.03	0.17
	standard deviation	0.07	0.08	0.09	0.08	0.09
	sum	0.46	0.84	0.66	1.00	5.17
